# Differentially Expressed Genes during Contrasting Growth Stages of *Artemisia annua* for Artemisinin Content

**DOI:** 10.1371/journal.pone.0060375

**Published:** 2013-04-03

**Authors:** Priya Nair, Amita Misra, Alka Singh, Ashutosh K. Shukla, Madan M. Gupta, Anil K. Gupta, Vikrant Gupta, Suman P. S. Khanuja, Ajit K. Shasany

**Affiliations:** CSIR-Central Institute of Medicinal and Aromatic Plants, Lucknow, Uttar Pradesh, India; Université Pierre et Marie Curie, France

## Abstract

*Artemisia annua* is the source of antimalarial phytomolecule, artemisinin. It is mainly produced and stored in the glandular secretory trichomes present in the leaves of the plant. Since, the artemisinin biosynthesis steps are yet to be worked out, in this investigation a microarray chip was strategized for the first time to shortlist the differentially expressing genes at a stage of plant producing highest artemisinin compared to the stage with no artemisinin. As the target of this study was to analyze differential gene expression associated with contrasting artemisinin content *in planta* and a genotype having zero/negligible artemisinin content was unavailable, it was decided to compare different stages of the same genotype with contrasting artemisinin content (seedling - negligible artemisinin, mature leaf - high artemisinin). The SCAR-marked artemisinin-rich (∼1.2%) Indian variety ‘CIM-Arogya’ was used in the present study to determine optimal plant stage and leaf ontogenic level for artemisinin content. A representative EST dataset from leaf trichome at the stage of maximal artemisinin biosynthesis was established. The high utility small scale custom microarray chip of *A. annua* containing all the significant artemisinin biosynthesis-related genes, the established EST dataset, gene sequences isolated in-house and strategically selected candidates from the *A. annua* Unigene database (NCBI) was employed to compare the gene expression profiles of two stages. The expression data was validated through semiquantitative and quantitative RT-PCR followed by putative annotations through bioinformatics-based approaches. Many candidates having probable role in artemisinin metabolism were identified and described with scope for further functional characterization.

## Introduction

*Artemisia annua* (Asteraceae) is the only source for important antimalarial phytomolecule “artemisinin”. This molecule, an endoperoxide sesquiterpene lactone [1] is active against multi-drug-resistant strains of the malarial parasite. In spite of the report on development of resistance against artemisinin [2], the phytomolecule remains a potent weapon in the arsenal against malaria. Since, it is difficult to synthesize artemisinin chemically and its production in cell/tissue culture is very low, the plant remains the only major source for the drug [3]. Artemisinin is detected in aerial parts of the plant, mainly in leaves, stem and inflorescence and little or none in roots or pollen, with the content varying from 0.01–1.2% in different genotypes of *A. annua* [4], [5], [6]. Low supply of artemisinin against high demand has provided major impetus for research on identification of genes and regulatory factors towards enhancing the *in planta* and/or heterologous production of the phytomolecule.

*In planta,* artemisinin is biosynthesized from isopentenyl pyrophosphate (IPP) *via* farnesyl pyrophosphate (FPP). FPP is converted to amorpha-4,11-diene by the action of amorpha-4,11-diene synthase (*ads*), the step commiting the metabolic flux towards artemisinin. The next reaction is a three step oxidation of amorpha-4,11-diene to artemisinic acid *via* formation of artemisinic alcohol and artemisinic aldehyde by a cytochrome P450 monooxygenase (*cyp71av1*) [7]. Recently two more genes, a double bond reductase (*dbr2*) [8] and an aldehyde dehydrogenase (*aldh1*) [9] presumably involved in artemisinin biosynthesis pathway, were also cloned and indicated in the conversion of artemisinic aldehyde to its dihydro form and then to dihydroartemisinic acid (DHAA), respectively. It is believed that the two bifurcated pathways generating either arteannuin B or artemisinin as end products compete with each other for precursors [10]. Wallaart *et al.* [11], [12] suggested DHAA as a reactive oxygen species (ROS) scavenger and is probably converted to artemisinin by more than one non-enzymatic, spontaneous photo-oxidation reactions [13]. Besides, there may be other unknown diversions from FPP affecting artemisinin biosynthesis. Also, most of the regulatory factors involved in artemisinin biosynthesis are yet to be elucidated. This necessitates searching for novel structural and regulatory genes involved in artemisinin biosynthesis in the plant. Based on this, ‘CIM-Arogya’, a SCAR-marked high yielding (∼1.2% artemisinin content on dry weight basis) variety of *A. annua* [5], [14], [15], similar in artemisinin content to the European variety ‘Artemis’, was analyzed in the present study. This variety is well accepted by the industry and grown extensively by farmers. Recently, the genetic map of *A. annua* has also been constructed and used to identify loci affecting yield of artemisinin [16]. EST-based [17] and deep transcriptome sequencing [18] approaches have been attempted in the plant earlier for identifying genes. These efforts will definitely facilitate new gene discovery and shed light on the regulatory mechanism of artemisinin metabolism and trichome function in *A. annua*. Still, there is a need to have a representative EST dataset from the trichome of elite Indian *A. annua* varieties like ‘CIM-Arogya’ to enrich the existing information helping to identify candidates for gene bioprospecting and downstream expression analysis.

Artemisinin content in the plant in various climates and regions has been studied in the past, mainly in China [19]. In the present study, the critical developmental/seasonal stages for the biosynthesis/accumulation of artemisinin were defined and a representative EST dataset from leaf trichome at the stage of maximal artemisinin biosynthesis was established. A high utility small scale (750 target genes) custom microarray chip of *A. annua* containing all the significant artemisinin biosynthesis-related genes was designed using the in-house established EST dataset and strategically selected candidates from the *A. annua* Unigene database (NCBI). The custom array was employed to compare the gene expression profiles of two plant developmental stages having contrasting metabolite (artemisinin) levels. The microarray data was validated through quantitative RT-PCR and differentially expressing candidates were identified in both the stages providing leads for further functional analysis *in planta*. This is the first report on microarray analysis for a limited selected genes in *A. annua*.

## Materials and Methods

### Plant Material and Growth Conditions

The seeds of *A. annua* (cv. CIM-Arogya) were obtained from the National Gene Bank for Medicinal and Aromatic Plants, CSIR-CIMAP, Lucknow and sown in earthen pots (20 cm high and 20 cm internal diameter) containing a mixture of soil and farmyard manure in the ratio of 1∶1. The plants were grown in glass house under standard conditions of light, temperature and humidity. For the field experiments (for chemo-profiling) the nursery (seedlings having 10 cm height) were transplanted in the field with spacing 50 cm between rows and 30 cm between plants and grown using standard agronomic procedures decribed earlier [14]. For the array-based experiment, the two contrasting stages were selected (six-day-old seedling and six-month-old mature plant).

### Chemo-profiling for Artemisinin

For *in planta* artemisinin content analysis, 0.1 g shade-dried leaves/seedlings were ground, boiled in hexane, filtered and evaporated. The residue was used for artemisinin analysis through HPTLC following the protocol described by Misra *et al.* [20]. All samplings for the artemisinin content analysis were carried out in triplicate for analysis.

### Construction of Leaf Glandular Trichome (GT) cDNA Library and EST Sequencing

GTs were isolated from 500 g leaves of a mature six-month old plant (at the stage of maximal artemisinin biosynthesis) using the protocol described by Teoh *et al.* [7]. For cDNA library construction, total RNA was isolated from the trichome-enriched leaf tissue according to Chomczynski and Sacchi [21] and poly (A)^+^ mRNA was isolated following the protocol of Shukla *et al.* [22]. For the microarray experiment, total RNA was isolated from seedling and mature plant leaf using RNeasy Mini Kit (Qiagen) according to manufacturer’s guidelines. RNA concentration was quantified by measuring absorbance at 260 and 280 nm by NanoDrop (NanoDrop, USA) and RNA quality was evaluated in Agilent 2100 Bioanalyzer (Agilent Technologies Inc., Palo Alto, CA). Total RNA meeting the quality standards was released for probe generation. cDNA library was prepared by using the ZAP Express® cDNA Synthesis Kit and packaged by Gigapack® III gold cloning kit (Stratagene, USA). The primary library was amplified to a titer of 1.6 x 10^7^ pfu/ml in *E. coli* strain XL1-Blue MRF’. This was mass excised using ExAssist™ helper phage and *E. coli* strain XL1 Blue MRF’ (at a MOI of 1∶10 lambda phage-to-cell ratio and 10∶1 helper phage-to-cells ratio) to obtain recombinant cDNA clones in pBK-CMV phagemid. The excised phagemids were titered using *E. coli* strain XLOLR and the recombinants were selected by blue/white screening on X-gal/IPTG-coated plates containing kanamycin (50 µg/ml). The clones from the amplified library (over 90% insert frequency) were used for end sequencing to generate a small in-house leaf trichome EST dataset. The ESTs were analyzed as described earlier [23].

### *A. annua* Custom Array Design and Preparation

A small scale microarray chip (with 750 target genes) was designed for *A. annua*. The sequences for *A. annua* were taken from three sources - *A. annua* Unigene (NCBI) unique sequence library (having 9462 entries), 385 CIMAP in-house *A. annua* (CIM-Arogya) EST sequences (Accession Numbers GT735932-GT736316) and an additional 22 in house cytochrome P450 (*cyp*) gene sequences (**Table S1**) [20]. The *A. annua* Unigene was searched with keywords – decarboxylase, kinase, reductase, reductoisomerase, regulator, synthase, synthetase, transcription, transcription factor, transferase, transporter, *etc* and a few candidates belonging to categories like cytochromes, transcription factors, transporters, reductases, synthases, dehydrogenases, peroxidases, isomerases and known genes from *A. annua* were shortlisted based on their putative involvement in artemisinin biosynthesis/metabolism/diversion pathways/regulation. From each Unigene cluster, a single representative EST-clone sequence provided for cluster by NCBI was included. This sequence is the longest and most homologous strand present within the cluster. This set of shortlisted candidates was then analyzed for redundancy with the in house sequences in the subsequent steps.

Clustering was carried out for the 385 in house ESTs using CAP3 to remove the redundant sequences, which resulted into 174 unigenes (59 contigs +115 singletons). Further the ESTs and their CAP3 cluster contigs, were annotated by using NCBI’s Local-BLAST’s tblastx functionality. This function compared the given sequences with the ‘*Arabidopsis*’ RefSeq database and a database of RefSeq sequences of other ‘Flowering Plants’ to indicate any putative homology found to annotated plant organism’s mRNA reference sequences. The program output files were analyzed using TblastParse (an internal report analysis program of Ocimum Biosolutions, India) and significant annotation retrieved and reported for each EST. The Unigene selection set was compared to in house sequences using NCBI’s Local-BLAST program to eliminate redundancy occurring between the two datasets. Four singletons (GT735961, GT736012, GT736179, and GT736228) and 4 *cyp* gene sequences [(CIM-Arog_CYP01 (KC594703), CIM-Arog_CYP04 (JN594505), CIM-Arog_CYP06 (GU318228), CIM-Arog_CYP08 (KC594704)] of in-house sequences were found to be already represented in the NCBI Unigene selections. CIM-Arog_CYP05 (JN594506) could not be spotted on the array. It was also ensured that inclusion of any sequence showing homology to ribosomal genes and targets for which oligos could not be designed were avoided. Finally, a total of 157 in house unigenes (55 contigs +102 singletons) and 17 *cyp* genes [20] were represented on the array apart from the other selections from the publicly available Unigenes to make a target number of 750 genes. After checking the cross hybridization of the designed 50 mer oligos, the custom *A. annua* array was prepared by Ocimum Biosolutions, Hyderabad, India and its platform data has been submitted to Gene Expression Omnibus (GEO, NCBI) under accession number GPL15698.

### Probe Generation, Hybridization and Analysis

A dual channel procedure with dye-swap arrangement was adopted in the study of the two samples - seedling and mature leaf, to compare the expression levels of target genes in seedling against mature leaf. The expression data was generated on 1,569 probes, with two replicates. Five microgram of total RNA was used for amplification with the help of Amino Allyl MessageAmp™ II aRNA Amplification Kit from Ambion by linear transcription based RNA amplification system to produce cRNA. Briefly, mRNA was reverse transcribed with an oligo (dT) primer bearing T7 promoter at 42°C for 2 h and second strand cDNA synthesis was carried out at 16°C for 2 h. The resulting cDNA was purified and transcribed with T7 RNA polymerase to generate multiple copies of aminoallyl antisense RNA (aRNA) at 37°C for 16 h. aRNA was then labelled with Cy3TM/Cy5TM post-labelling reactive dye pack (GE Healthcare, UK) at room temperature and unincorporated Cy3/Cy5 molecules were removed by purification process using QIAGEN PCR purification kit before hybridization. Ten microgram of the labeled aRNA in 75 µl of Ocimum’s Hyb buffer was used for hybridization with the *A. annua* custom array chip. Hybridized chips were scanned using Affymetrix 428TM Array Scanner at three different PMT gains (40, 50 and 60) and the data was analyzed using Genowiz software (Ocimum Biosolutions, Hyderabad). Image analysis was carried out using Imagene, version 5.6.1. The signal values obtained at the three PMT settings were averaged, to get the signal mean for further analysis. Replicate genes were also averaged before normalization. Signal values obtained from each channel were log_2_ transformed and normalized using LOWESS algorithm and median absolute deviation (MAD) scaling. For each sample, Cy3 and Cy5 intensities were averaged, and used to compare the samples. During normalization, paired slide dye-swap method was followed to overcome the dye-bias during the comparison and then MAD was performed to adjust data into same scale [24]. The normalized (adjusted) data was subsequently used in differential expression (DE) analysis, which was performed using fold change technique. The data from this microarray experiment has been submitted to GEO (NCBI) under series accession number GSE39098 [associated sample data GSM956130 (leaf *vs* seedling) and GSM956131 (seedling *vs* leaf)]. The ‘OBSca028_’ prefix in the gene/probe IDs in the submitted data has been abbreviated here as ‘Aa’ for convenience.

### Semiquantitative and Quantitative RT-PCR Analysis for Validation

The gene expression profiles obtained in the microarray analysis were validated through semiquantitative and quantitative RT-PCR. For semiquantitative RT-PCR, the total RNA was isolated from the six-day-old seedling and six-month-old mature plant leaf of *A. annua* using TRIzol® reagent (Invitrogen, USA). The quality and quantity of total RNA was assessed through ethidium bromide staining as well as Nanodrop ND1000 spectrophotometer. Equal amount (4 µg) of DNaseI-treated total RNA was used for first strand cDNA synthesis by Thermoscript RT-PCR System (Invitrogen). *A. annua* actin (EU531837) was used as a control for the gel-based semiquantitative analysis of gene expression. The primer sequences used for the semi-quantitative RT-PCR-based validation of selected target genes were designed using Gene Runner *version 3.05* (Hastings Software, Inc.) and are listed in **Table S2**. The gene expression profiles of known genes of *A. annua*, mainly belonging to the artemisinin (and other sesquiterpene) biosynthetic pathway were validated for trichomes isolated from leaf through TaqMan chemistry-based Real Time PCR (**Table S3**). The level of gene expression was analyzed following the protocol described by Misra *et al.* [20] and finally log_10_ RQ values were calculated and represented.

## Results

### Ontogeny and Plant Age-related Variation in Artemisinin Content

**Figure 1:**
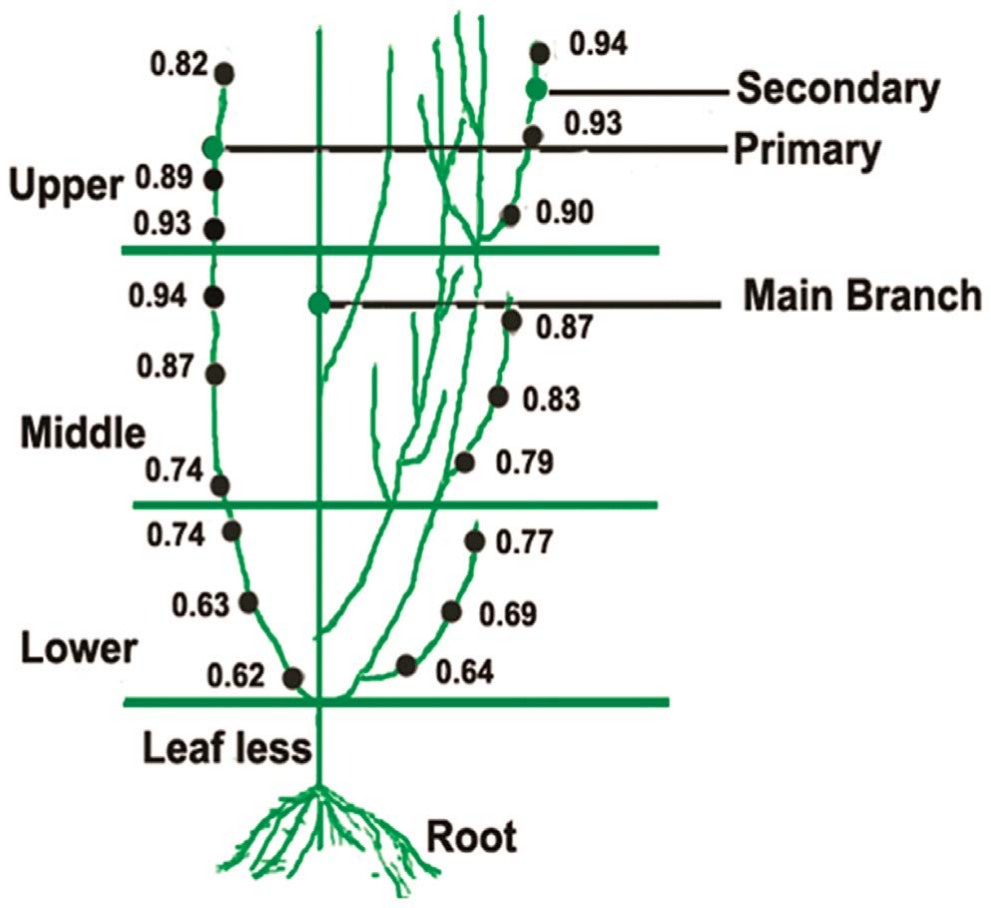
A line diagram showing variation in artemisinin content in leaves at various ontogenic levels of the *A. annua* plant. Sampling was carried out in triplicate, and standard deviation ranged between 1.5–12.3% of mean values.

**Figure 2:**
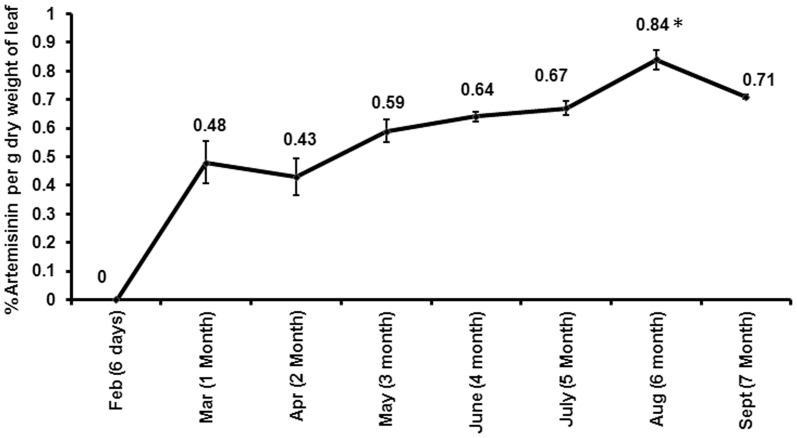
Developmental and temporal variation in leaf artemisinin content of *A. annua* through its cropping season. Y axis represents % artemisinin content per g dry weight of young leaves at upper levels of secondary branches. Sampling was carried out in triplicate.

Experiment was carried out to study the ontogenic variation of artemisinin content in mature (6-month-old) field grown *A. annua* (**Figure 1**). The aerial portion of the plant was demarcated into upper (top about 30 cm), middle (about 30 cm), lower (between middle and leafless region, about 30 cm) and leafless (upto 45 cm height from the ground) regions. Leaf samples were collected from different ontogeny levels of primary and secondary branches as indicated in **Figure 1**. Artemisinin content was always found to be optimum in the young leaves at upper levels of secondary branches. The content of artemisinin in the stem, seed and seed husk was found to be 1/10^th^, 1/35^th^ and 1/3^rd^ respectively as compared to the leaves, whereas it was undetected in the roots of the plant (data not shown). Another related experiment was carried out to study the developmental variation of leaf artemisinin content in the plants under north Indian conditions (**Figure 2**). Starting from February (6-day-old seedling stage), monthly sampling of leaves was carried out till September. Leaf artemisinin content was found to increase from a value “undetected” at the six-day seedling stage, reaching the maximum at the pre-flowering stage (6-month-old plant/August) and declining thereafter.

### Representative ESTs from Metabolically Active Leaf GTs

After defining the optimal plant stage and ontogenic level for highest leaf artemisinin content through chemo-profiling, a leaf trichome cDNA library was constructed. Around 500 cDNA clones were sequenced generating 458 ESTs and were submitted to dbEST (under accession numbers: GT735901–GT735931, GT735932–GT736316, GT736317–GT736358). More ESTs were not generated as ∼ 85000 ESTs (as well as unigenes) of *A. annua* became available in the NCBI public database. Hence, strategic selection of candidates [(genes/ESTs (including unigenes)] from the NCBI database and in house generated dataset was opted for limited array-based gene expression profiling in plant developmental stages contrasting for their artemisinin content.

### Comparative Gene Expression Analysis

**Figure 3:**
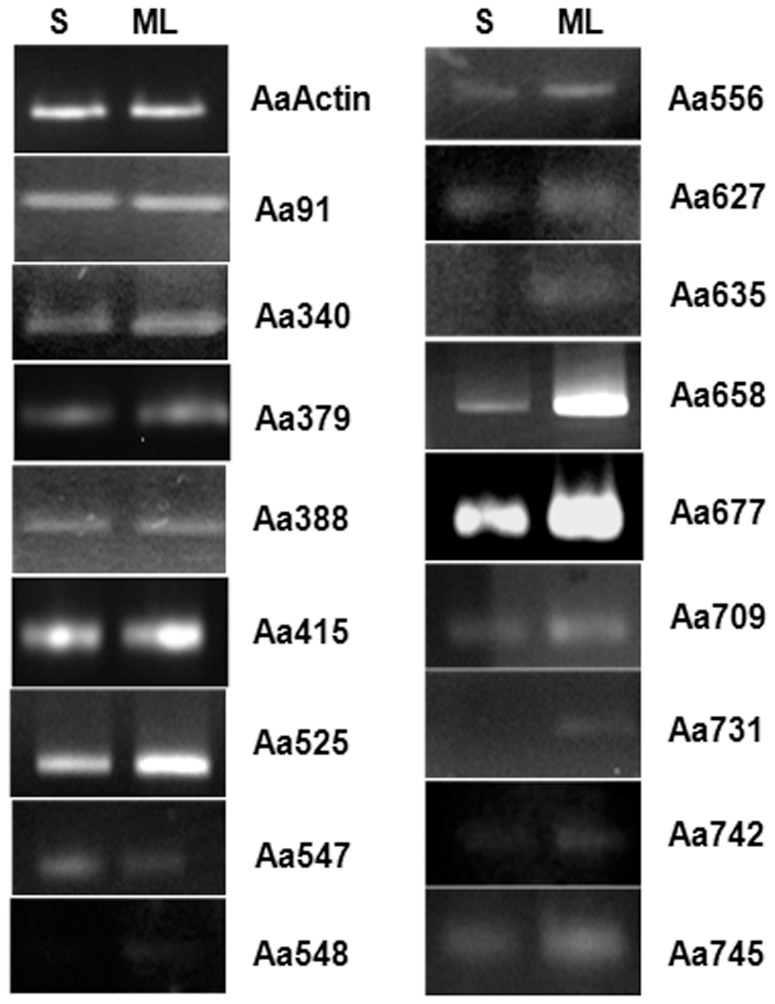
Representative gel image depicting the semiquantitative RT-PCR-based validation of *A. annua* microarray data (seedling *vs* mature leaf) for the differentially expressed genes in the plant stages (seedling – negligible artemisinin; mature leaf – high artemisinin) contrasting for artemisinin content.

**Figure 4:**
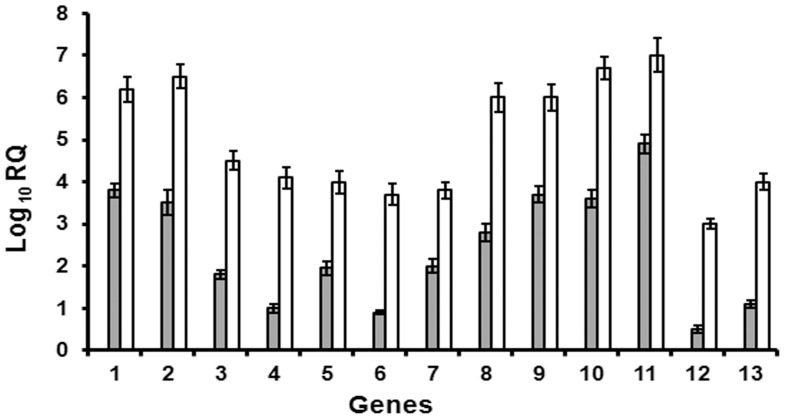
Comparison of quantitative gene expression levels at seedling and mature leaf stage of *A. annua*. Expression of all the genes in 6-day-old seedling (calibrator) was equilibrated to “0”. White bars represent expression in the leaf of 3-month-old plant and solid bars in 6-month-old plant. Y-axis represents Log_10_ RQ, where relative quantity (RQ) equilibrating the expression in seedling is 1RQ value. Data represent mean±standard error of at least 3 biological replicates. The genes studied are 1. Farnesyl pyrophosphate synthase (Aa398); 2. Cytochrome P450 reductase (CPR) (Aa15); 3. Amorpha-4,11-diene synthase (Aa408); 4. P450 monooxygenase (CYP71) (Aa561); 5. Linalool synthase (Aa442); 6. Germacrene-A synthase (Aa434); 7. Squalene synthase (Aa407); 8. β–Caryophyllene synthase (Aa417); 9. Sesquiterpene cyclase (Aa560); 10. 1-Deoxy-D-xylulose-5-phosphate reductoisomerase (DXR) (Aa554); 11. HMG-CoA reductase (Aa689).

Selected 385 ESTs (GT735932–GT736316) from the in house generated dataset, shortlisted “Unigene” candidates from NCBI and in house generated cyp genes were taken up for custom array-based comparative gene expression analysis in mRNA populations derived from two contrasting plant developmental stages (6-day-old seedling - negligible artemisinin, mature leaf from 6-month-old plants - high artemisinin). For the comparative analysis, genes with log fold change value≥0.5849 (FC≥1.5) were assumed as up-regulated while genes with log fold change ≤−0.5849 (FC≤0.66) were assumed as down-regulated in seedling, compared to mature leaf sample taken as the control. Based on this criterion, of 750 target genes on the chip, 158 genes were found to be down-regulated (**Table S4**), whereas 73 genes were up-regulated in seedling (**Table S5**) compared to mature leaf. The heat maps for the differentially expressing genes is provided in **Figures S1** (upregulated in seedling) and **S2** (downregulated in seedling). However, while selecting the genes for downstream analysis and usage, a more stringent threshold was used, where genes with log fold change value≥1 (FC≥2) were considered to be up-regulated and those with log fold change value≤−1 (FC≤0.50) were considered to be down-regulated (with a few exceptions like the known genes/ESTs of *A. annua*). On the basis of this stringent criterion, 98 genes qualified for the “down-regulated in seedling” category and 27 for the “up-regulated in seedling” category (**Table 1**). The results obtained in the microarray experiment were verified through semiquantitative RT-PCR analysis for a representative set of genes (**Figure 3**) and through TaqMan chemistry-based Real Time PCR for the known pathway genes (**Figure 4**).

**Table 1:** Differentially expressed genes in high (mature leaf) and low (seedling) artemisinin stages of *A. annua*.

S. No.	Gene/Probe ID	Gene/Probe Homology-based Annotation/Description	Log Fold Change	Fold Change	*Tissue specificity
**Up-regulated in seedling**					
1.	Aa19	Cytochrome P450, family 51 (sterol 14-demethylase) [*Arabidopsis thaliana*] (NP_172633)	0.968547	1.956868	T
2.	Aa28	Cytochrom P450-like protein [*Arabidopsis thaliana*] (AAM66094)	2.027971	4.07831	L
3.	Aa31	Cytochrome P450, family 77, subfamily B, polypeptide 1 [*Arabidopsis thaliana*] (NP_172626)	1.060499	2.085653	L
4.	Aa37	Cytochrome P450, family 72, subfamily A, polypeptide 8 [*Arabidopsis thaliana*] (NP_188080)	1.193271	2.286707	T/L
5.	Aa67	Putative cytochrome P450 [*Arabidopsis thaliana*] (NP_001117558)	1.102678	2.14753	L
6.	Aa84	Cytochrome P450, family 81, subfamily D, polypeptide 8 [*Arabidopsis thaliana*] (NP_195453)	2.064044	4.181568	L
7.	Aa94	Nuclear transcription factor Y subunit C-1 [*Arabidopsis thaliana*] (NP_190428)	1.270854	2.413043	L
8.	Aa127	AP2/ERF and B3 domain-containing transcription factor RAV1 [*Arabidopsis thaliana*] (NP_172784)	1.283591	2.434441	L
9.	Aa150	RGA1 protein [*Arabidopsis thaliana*](CAA72177)	1.179778	2.26542	T
10.	Aa158	Indoleacetic acid (IAA)-inducible gene (IAA7) [*Arabidopsis thaliana*] (AAM65301)	1.595321	3.021618	T
11.	Aa185	Transcription factor AS1 [*Arabidopsis thaliana*] (NP_181299)	1.214433	2.320496	L
12.	Aa216	Ethylene-responsive transcription factor ERF025 [*Arabidopsis thaliana*] (NP_200015)	1.396486	2.632596	T
13.	Aa296	Auxin efflux carrier component 3 [*Arabidopsis thaliana*] (NP_177250)	0.965973	1.953381	L
14.	Aa322	Enoyl-ACP reductase [*Arabidopsis thaliana*] (CAA74175)	1.145828	2.212731	L
15.	Aa346	Rossmann-fold NAD(P)-binding domain containing protein [*Arabidopsis thaliana*] (NP_849428)	1.087424	2.124942	L
16.	Aa436	Tryptophan synthase alpha chain [*Arabidopsis thaliana*] (NP_192170)	1.074609	2.106151	L
17.	Aa445	1-aminocyclopropane-1-carboxylate synthase 6 [*Arabidopsis thaliana*] (NP_192867)	1.114773	2.165609	T
18.	Aa476	Dihydrolipoyllysine-Residue Succinyltransferase Component Of 2-Oxoglutarate Dehydrogenase Complex 1 [*Arabidopsis Thaliana*] (Np_200318)	1.087914	2.125665	L/T
19.	Aa488	UDP-glucose dehydrogenase, putative [*Arabidopsis thaliana*] (AAM67208)	0.959374	1.944466	T
20.	Aa508	Pyruvate dehydrogenase E1 component subunit alpha [*Arabidopsis thaliana*] (NP_171617)	1.41223	2.661482	T
21.	Aa547	Peroxidase ATP24a [*Arabidopsis thaliana*] (CAA72484)	1.827388	3.54894	T
22.	Aa554	*A. annua* 1-deoxy-D-xylulose-5-phosphate reductoisomerase (DXR1) mRNA, complete cds (AF182287)	1.349311	2.547904	T
23.	Aa555	*A. annua* mRNA for putative sesquiterpene cyclase (cASC125 gene) (AJ271792)	1.543833	2.915681	NA
24.	Aa590	Cytochrome P450, family 77, subfamily B, polypeptide 1 [*Arabidopsis thaliana*] (NP_172626)	1.20053	2.298241	NA
25.	Aa600	Maturase K [*Arabidopsis thaliana*] (AAG43340)	1.900507	3.733445	NA
26.	Aa718	Phosphoenolpyruvate carboxykinase [ATP] [*Arabidopsis thaliana*] (NP_195500)	0.779621	1.716679	NA
27.	Aa738	Catalase 2 [*Arabidopsis thaliana*] (NP_001031791)	1.093858	2.134441	NA
**Down-regulated in seedling**					
**S. No.**	**Gene/Probe ID**	**Gene/Probe Homology-based Annotation/Description**	**Log Fold Change**	**Fold Change**	
1.	Aa15	*A. annua* cytochrome P450 reductase (EF197890)	−1.378634	0.384583	T/L
2.	Aa21	*A. annua* putative steroid 23-alpha-hydroxylase cytochrome P450 mRNA(DQ363132)	−0.97876	0.507416	T
3.	Aa25	*A. annua* putative taxadiene 5-alpha-hydroxylase cytochrome P450 mRNA, (DQ363134)	−3.832573	0.070191	L
4.	Aa26	Cytochrome b5 isoform 1 [*Arabidopsis thaliana*] (NP_200168)	−1.55401	0.340562	L/T
5.	Aa27	Cytochrome b5 isoform 1 [*Arabidopsis thaliana*] (NP_200168)	−2.297156	0.203464	L
6.	Aa29	Abscisic acid 8′-hydroxylase 2 [*Arabidopsis thaliana*] (NP_180473)	−1.358201	0.390068	L
7.	Aa30	Cytochrome b5 isoform 1 [*Arabidopsis thaliana*] (NP_200168)	−0.932884	0.52381	L/T
8.	Aa32	Cytochrome P450, family 72, subfamily A, polypeptide 15 [*Arabidopsis thaliana*] (NP_188087)	−1.072802	0.475395	L
9.	Aa33	Cytochrome b561 [*Arabidopsis thaliana*] (AAM62824)	−1.54308	0.343152	L/T
10.	Aa51	*A. annua* putative flavonoid 3 -hydroxylase cytochrome P450 mRNA, (DQ363131)	−1.50153	0.353179	L
11.	Aa53	*A. annua* putative taxane 13-alpha-hydroxylase cytochrome P450 mRNA, (DQ363133)	−0.840187	0.558571	NA
12.	Aa91	Multiprotein bridging factor 1A [*Arabidopsis thaliana*] (NP_565981)	−1.652788	0.318025	T
13.	Aa93	Similar to Schizosaccharomyces CCAAT-binding factor. [*Arabidopsis thaliana*] (AAB70410)	−1.211259	0.431892	L/T
14.	Aa111	Homeobox protein knotted-1-like 7 [*Arabidopsis thaliana*] (NP_564805)	−1.488941	0.356274	L
15.	Aa138	NAC domain containing protein 83 [*Arabidopsis thaliana*] (NP_196822)	−1.225093	0.42777	T/L
16.	Aa141	DELLA protein RGA [*Arabidopsis thaliana*] (NP_178266)	−0.952092	0.516882	L
17.	Aa143	BEL1-like homeodomain 1 [*Arabidopsis thaliana*] (AAK43836)	−2.702299	0.153648	L
18.	Aa163	Auxin-responsive protein IAA9 [*Arabidopsis thaliana*] (NP_569017)	−1.3361	0.39609	L
19.	Aa167	Transcription factor ILR3 [*Arabidopsis thaliana*] (NP_200279)	−1.033723	0.488448	L
20.	Aa171	RNA polymerase sigma factor [*Arabidopsis thaliana*] (NP_197800)	−3.68014	0.078013	T/L
21.	Aa206	Transcription factor TCP7 [*Arabidopsis thaliana*] (NP_197719)	−1.629547	0.32319	L
22.	Aa207	Indole-3-acetic acid inducible 4 [*Arabidopsis thaliana*] (ADC29372)	−1.078584	0.473493	T
23.	Aa226	Basic leucine-zipper 44 [*Arabidopsis thaliana*] (NP_177672)	−1.262889	0.416709	L
24.	Aa233	Protein agamous-like 42 [*Arabidopsis thaliana*] (NP_568952)	−2.832735	0.140366	T
25.	Aa241	Putative AP2 domain transcriptional regulator, 5′ partial; 1-558 [*Arabidopsis thaliana*] (AAG52091)	−2.208228	0.2164	T
26.	Aa244	NAC domain containing protein 83 [*Arabidopsis thaliana*] (NP_196822)	−1.485032	0.357241	T
27.	Aa247	Nuclear factor Y, subunit C13 [*Arabidopsis thaliana*] (NP_199139)	−1.266693	0.415611	L
28.	Aa266	Scarecrow-like protein 30 [*Arabidopsis thaliana*] (NP_001078251)	−1.359046	0.38984	L
29.	Aa268	Two-component response regulator-like APRR3 [*Arabidopsis thaliana*] (NP_001190575)	−1.127476	0.457716	L
30.	Aa271	Homeodomain-like transcriptional regulator [*Arabidopsis thaliana*] (NP_001190470)	−1.390764	0.381363	L
31.	Aa275	Two-component response regulator-like APRR5 [*Arabidopsis thaliana*] (Q6LA42)	−1.223519	0.428237	T
32.	Aa285	Phosphate transporter [*Arabidopsis thaliana*] (BAE98881)	−3.1146	0.115455	L/T
33.	Aa289	Putative Mn-specific cation diffusion facilitator transporter [*Arabidopsis thaliana*] (NP_181477)	−2.310703	0.201562	L
34.	Aa316	*A. annua* voucher huayang 2 12-oxophytodienoate reductase-like protein mRNA, (EU848577)/*A. annua* artemisinic aldehyde delta-11(13) reductase (ACH61780.1)	−1.006099	0.497891	T
35.	Aa335	Tropine dehydrogenase [*Arabidopsis thaliana*] (NP_196225)	−1.516377	0.349563	L
36.	Aa340	Putative AX110P protein [*Arabidopsis thaliana*] (AAK76525)	−1.328382	0.398215	L
37.	Aa347	Clavaminate synthase-like protein [*Arabidopsis thaliana*] NAD(P)-linked oxidoreductase-like protein [*Arabidopsis thaliana*] (NP_188773)	−1.137866	0.454431	L
38.	Aa372	Thioredoxin-like protein [*Arabidopsis thaliana*] (NP_030274)	−2.248172	0.210491	L
39.	Aa379	Protein SRG1 [*Arabidopsis thaliana*] (NP_173145)	−1.486589	0.356855	L
40.	Aa388	*A. annua* IPP/DMAPP synthase (ABY57296.1)	−1.83161	0.280951	T/L
41.	Aa393	Ketol-acid reductoisomerase [*Arabidopsis thaliana*] (AAW82381)	−1.348233	0.392773	L/T
42.	Aa396	Male sterility 2-like protein [*Arabidopsis thaliana*] (CAA20592)	−1.075108	0.474636	L
43.	Aa401	*A. annua* beta-amyrin synthase (BAS) mRNA, complete cds (EU563939)	−0.991458	0.502969	L
44.	Aa407	*A. annua* squalene synthase mRNA, complete cds (AF302464)	−1.10467	0.465009	L
45.	Aa408	*A. annua* mRNA for amorpha-4,11-diene synthase (kcs12 gene) (AJ251751)	−2.1082	0.231936	T
46.	Aa409	*A. annua* IPP/DMAPP synthase (ispH) mRNA, complete cds (EU332141)	−2.447293	0.183354	T/L
47.	Aa413	*A. annua* 8-epicedrol synthase (Ecs1) mRNA, complete cds (AF157059)	−1.725299	0.302436	L
48.	Aa415	Similar to dihydroflavonol reductase [*Arabidopsis thaliana*] (AAK68820)	−1.413989	0.375273	L/T
49.	Aa417	*A. annua* mRNA for putative sesquiterpene cyclase (cASC34 gene) (AJ271793)	−1.087717	0.470505	L
50.	Aa430	2C-methyl-D-erythritol 2,4-cyclodiphosphate synthase [*Arabidopsis thaliana*] (AAM62786)	−1.230706	0.426109	L
51.	Aa432	Mutant protein of chalcone synthase [*Arabidopsis thaliana*] (BAD89854)	−0.95799	0.514773	L/T
52.	Aa442	*A. annua* (3R)-linalool synthase (QH5) mRNA, complete cds (AF154124)	−2.933513	0.130895	T
53.	Aa443	*A. annua* (3R)-linalool synthase (QH1) mRNA,complete cds (AF154125)	−1.907274	0.266596	NA
54.	Aa446	flavone synthase [*Arabidopsis thaliana*] (CAP09039)	−1.636486	0.321639	L/T
55.	Aa456	*A. annua* (-)-beta-pinene synthase (QH6) mRNA, complete cds (AF276072)	−1.201715	0.434758	NA
56.	Aa483	3-hydroxyisobutyrate dehydrogenase [*Arabidopsis thaliana*] (NP_567617)	−1.020856	0.492824	L/T
57.	Aa525	L-ascorbate peroxidase [*Arabidopsis thaliana*] (NP_172267)	−1.78779	0.289615	L
58.	Aa528	Peroxidase ATP1a [*Arabidopsis thaliana*] (CAA66862)	−1.049413	0.483165	L/T
59.	Aa530	Peroxidase 3 [*Arabidopsis thaliana*] (NP_172018)	−2.260808	0.208655	L
60.	Aa540	Peroxidase 12 [*Arabidopsis thaliana*] (NP_177313)	−1.601021	0.329644	L/T
61.	Aa542	L-ascorbate peroxidase [*Arabidopsis thaliana*] (NP_172267)	−1.263772	0.416454	L
62.	Aa548	Peroxidase 52 [*Arabidopsis thaliana*] (NP_196153)	−2.091811	0.234586	L
63.	Aa556	Vacuolar-processing enzyme alpha-isozyme [*Arabidopsis thaliana*] (NP_180165)	−2.223755	0.214083	L/T
64.	Aa557	*A. annua* amorpha-4,11-diene monooxygenase (cyp71av1) mRNA, complete cds (DQ315671)	−1.320268	0.40046	NA
65.	Aa561	*A. annua* P450 monooxygenase (CYP71) mRNA, complete cds (DQ667171)	−2.119147	0.230183	NA
66.	Aa574	Chalcone isomerase [*Arabidopsis thaliana*] (AAA32766)	−1.631408	0.322773	L
67.	Aa576	cinnamate-4-hydroxylase [*Arabidopsis thaliana*] (CAP08828)	−1.141436	0.453308	NA
68.	Aa578	Flavonoid 3′-monooxygenase [*Arabidopsis thaliana*] (NP_196416)	−1.444084	0.367525	NA
69.	Aa580	Cytochrome P450, family 72, subfamily A, polypeptide 7 [*Arabidopsis thaliana*] (NP_188079)	−2.446256	0.183486	NA
70.	Aa582	Cytochrome P450 81F1 [*Arabidopsis thaliana*] (AAL69519)	−3.762036	0.073708	NA
71.	Aa583	Cytochrome like protein [*Arabidopsis thaliana*] (CAB16768)	−1.28472	0.410451	NA
72.	Aa584	Cytochrome P450, family 81, subfamily D, polypeptide 2 [*Arabidopsis thaliana*] (NP_195452)	−2.920529	0.132079	NA
73.	Aa589	Cytochrome P450, family 82, subfamily C, polypeptide 2 [*Arabidopsis thaliana*] (NP_194925)	−2.58676	0.166459	NA
74.	Aa592	Cytochrome P450, family 82, subfamily G, polypeptide 1 [*Arabidopsis thaliana*] (NP_189154)	−4.440151	0.046066	NA
75.	Aa627	Uncharacterized protein [*Arabidopsis thaliana*] (NP_001154473)	−2.758683	0.147759	NA
76.	Aa633	Cytochrome c oxidase subunit 3 [*Arabidopsis thaliana*] (NP_178782)	−0.963245	0.512902	NA
77.	Aa634	ATPase subunit 6 [*Arabidopsis thaliana*] (P92547)	−2.337004	0.197921	NA
78.	Aa635	Ethylene-responsive transcription factor 9 [*Arabidopsis thaliana*] (NP_199234)	−1.199513	0.435422	NA
79.	Aa653	Cytochrome c oxidase subunit 1 [*Arabidopsis thaliana*] (NP_085587)	−1.424429	0.372567	NA
80.	Aa658	No significant similarity	−1.815483	0.284109	NA
81.	Aa677	Unknown protein [*Arabidopsis thaliana*] (AAL32564)	−0.936743	0.522411	NA
82.	Aa689	*A. annua* 3-hydroxy-3-methylglutaryl-coenzyme A reductase 2 (AAA68965.1)	−0.99942	0.500201	NA
83.	Aa691	Retroelement pol polyprotein-like [*Arabidopsis thaliana*] (BAB10790)	−1.009578	0.496692	NA
84.	Aa694	Mannose-binding lectin-like protein [*Arabidopsis thaliana*] (NP_849691)	−1.962402	0.256601	NA
85.	Aa701	VQ motif-containing protein [*Arabidopsis thaliana*] (NP_001117300)	−0.986404	0.504734	NA
86.	Aa702	AAA ATPase containing von Willebrand factor type A domain-containing protein [*Arabidopsis thaliana*] (NP_176883)	−1.620919	0.325128	NA
87.	Aa709	Phospholipid hydroperoxide glutathione peroxidase-like protein [*Arabidopsis thaliana*] (BAA24226)	−1.471459	0.360617	NA
88.	Aa711	S-adenosylmethionine synthetase [*Arabidopsis thaliana*] (AAA32868)	−2.396271	0.189955	NA
89.	Aa714	Inorganic carbon transport protein-related protein [*Arabidopsis thaliana*] (NP_177233)	−1.022893	0.492129	NA
90.	Aa717	N-glyceraldehyde-2-phosphotransferase-like [*Arabidopsis thaliana*] (BAA97552)	−1.169467	0.444586	NA
91.	Aa731	L-ascorbate oxidase [*Arabidopsis thaliana*] (NP_001154729)	−4.713993	0.038102	NA
92.	Aa732	ATP sulfurylase like protein [*Arabidopsis thaliana*] (BAD95100)	−1.616018	0.326235	NA
93.	Aa741	Hypothetical protein [*Arabidopsis thaliana*] (BAD93976)	−2.299193	0.203177	NA
94.	Aa742	Putative serine/threonine kinase [*Arabidopsis thaliana*] (BAC43390)	−1.65833	0.316806	NA
95.	Aa745	Exosome complex component RRP4 [*Arabidopsis thaliana*] (NP_171835)	−2.140791	0.226755	NA
96.	Aa747	S-adenosyl-L-methionine-dependent methyltransferase-like protein [*Arabidopsis thaliana*] (NP_176478)	−1.808345	0.285518	NA
97.	Aa755	NmrA-like negative transcriptional regulator family protein [*Arabidopsis thaliana*] (NP_195634)	−2.839433	0.139716	L
98.	Aa757	NmrA-like negative transcriptional regulator family protein [*Arabidopsis thaliana*] (NP_195634)	−1.728272	0.301813	L

Criteria for the comparative analysis adopted here was that genes with log fold change≤−1 (FC≤0.50) were considered as down-regulated and those with log fold change value≥1 (FC≥2) were considered as up-regulated in seedling, whereby mature leaf sample was taken as the control. However, a few exceptions were there based on the perceived importance of the gene function in relation to the differences in the two plant stages.

* Tissue specificity is based on the available Unigene (NCBI) data. It refers to the approximate gene expression pattern as inferred from EST counts. However, for various reasons, EST counts may not be a true indication of gene activity. T = ESTs found in trichome only; L = ESTs found in leaf only; T/L = ESTs found in both trichome as well as leaf but predominantly in the trichome; L/T = ESTs found in both trichome as well as leaf but predominantly in the leaf; NA = Tissue specificity data not available.

## Discussion

### Optimal Leaf Ontogeny and Plant Age for Maximal *in Planta* Artemisinin Biosynthesis

Artemisinin is biosynthesized and stored in glandular trichomes (GT) of flowers and on both the surfaces of leaves [25], [26], [27]. In the genus *Artemisia*, the differentiation of foliar cells into GT cells is completed in a very young primordial stage of the leaf [25]. According to Lommen *et al.* [28], GT densities are highest at the young leaf stage and decreases after attaining maximal size. However, the number remains more or less constant till the maximal size, after which GT number decreases rapidly suggesting the rupture of GT over time in the older leaves. Similar decrease in the GT number in *Mentha arvensis* from upper expanding young, to the lower level leaves proceeding for senescence is reported [29]. Also, artemisinin concentrations are reported to be higher in upper leaves compared to lower in a branch [30], [31]. In the present study artemisinin content was found to be maximal in the top level leaves of the branches (**Figure1**). The leaf artemisinin content was found to increase from an undetectable value at the six-day seedling stage, reaching the maximum at the pre-flowering stage (6-month-old plant/August) and declining thereafter. This was also in consonance to the results obtained by Gupta *et al.* [31], Liersch *et al.* [32] and Zhang *et al.* [19]. These results indicated the stages of active GTs in the leaves biosynthesizing high artemisinin and leaf samples were collected for EST and hybridization analysis at this stage.

### Differential Gene Expression

Although a subtractive hybridization-based approach has been followed in *A. annua* earlier to compare blooming flowers and flower buds [33], the present study was more elaborate and comprehensively planned. The target of the present study was to analyze differential gene expression associated with contrasting artemisinin content *in planta*. Since an *A. annua* genotype having zero/negligible artemisinin content was unavailable, it was decided to compare different stages of the same genotype (CIM-Arogya) having contrasting artemisinin content. So, the seedling stage (negligible artemisinin) and mature plant leaf (high artemisinin) derived mRNA populations were used to analyze the custom *A. annua* array. Though, some noise in the data due to other variations in the samples (like developmental age related differences) cannot be ruled out, this was the best option available to compare high and low artemisinin content related gene expression profiles in the plant.

**Figure 5:**
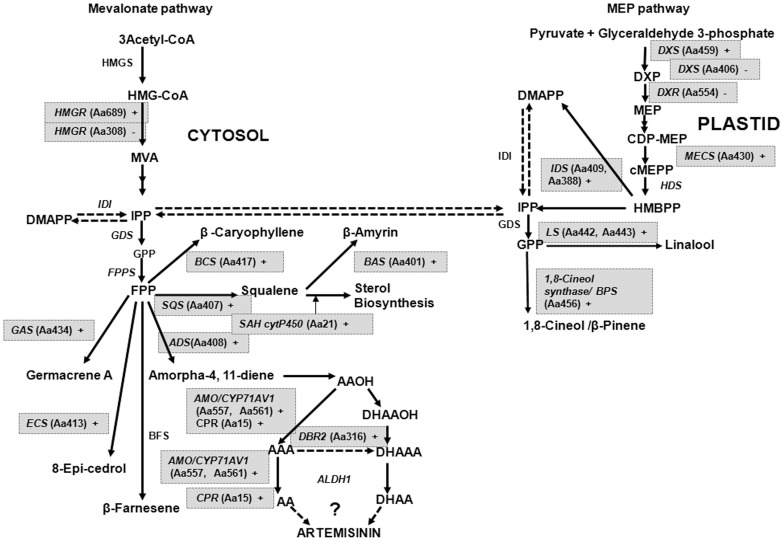
Mapping of the genes expressing differentially in seedling *vs* mature leaf on the artemisinin biosynthetic pathway in *A. annua*. Gene IDs are represented in parentheses. ‘+’ indicates up-regulated in mature leaf and ‘−’ indicates down-regulated in mature leaf as compared to seedling. The mapped genes from this study are highlighted. ADS: amorpha-4,11-diene synthase; ALDH1: aldehyde dehydrogenase 1; AMO/CYP71AV1: amorpha-diene oxidase; AAOH: artemisinic alcohol; AAA: artemisinic aldehyde; AA: artemisinic acid; BAS: β-amyrin synthase; BFS: β-farnesene synthase; CPR: cytochrome P450 reductase; BCS: β-caryophyllene synthase; CYP71AV1: amorphadiene-12-hydroxylase; DBR2: artemisinic aldehyde Δ11(13) reductase; DHAAOH: dihydroartemisinic alcohol; DHAAA: dihydroartemisinic aldehyde; DHAA: dihydroartemisinic acid; ECS: epi-cedrol synthase; DMAPP: dimethylallyl pyrophosphate; FPPS: farnesyl diphosphate synthase; FPP: farnesyl pyrophosphate; GAS: germacrene-A synthase; GPP: geranyl diphosphate; HMGR: 3-hydroxy-3-methyl-glutaryl coenzyme A reductase; HMGS: 3-hydroxy-3-methyl-glutaryl coenzyme A synthase; IPP: isopentenyl pyrophosphate; IDS: IPP/DMAPP synthase; MVA: mevalonic acid; SAH: steroid 23-alpha-hydroxylase; SQC: sesquiterpene cyclase; SQS: squalene synthase; BPS: β-pinene synthase; CDP-MEP: 4-cytidine 5′-diphospho-2-*C*-methyl-D-erythritol 2-phosphate; cMEPP: 2-C-methyl-D-erythritol 2,4-cyclophosphate; DXP: 1-deoxy-D-xylulose 5-phosphate; DXR: 1-deoxy-D-xylulose-5-phosphate reductoisomerase; DXS: 1-deoxy-D-xylulose-5-phosphate synthase; GPP: geranyl diphosphate; GDS: geranyl diphosphate synthase; HDR: hydroxy-2-methyl-2-(E)-butenyl 4-diphosphate reductase; HDS: hydroxy-2-methyl-2-(E)-butenyl 4-diphosphate synthase; HMBPP: 1-hydroxy-2-methyl-2-(E)-butenyl 4-diphosphate; LS: Linalool synthase; MECS: 2-C-methyl-D-erythritol 2,4-cyclodiphosphate synthase; MEP: 2-C-methyl-D-erythritol 4-phosphate.

Thirty six candidates showing significant fold change in expression in the two contrasting plant stages were identified for further gene prospecting (**Table S2**). **Figure 3** depicts representative genes that were taken up for validation through semi-quantitative RT-PCR. The secondary metabolism related genes were specifically validated for the trichome expression (**Figure 4**) as these are reported to be expressing in GTs present on both surfaces of the leaf. The increasing trend in expression was similar for all the genes as the plant matured from 6-day-old seedling to 6-month stage. The selection of the genes for microarray was also based on the relevance of their annotated function to the objective of association with artemisinin metabolism. Many genes found to be directly or indirectly influencing terpenoid (specially artemisinin) biosynthesis were found to be differentially expressed in the seedling as compared to the mature leaf and could be mapped on the pathway (**Figure 5**).

Expectedly, most of the known artemisinin (as well as other sesquiterpene) metabolism-related genes were found to be having higher expression at transcript level in the mature leaf as compared to the seedling stage. These included candidates like cytochrome P450 reductase (Aa15), squalene synthase (Aa407), artemisinin aldehyde delta-11(13) reductase (Aa316), isopentenyl pyrophosphate/dimethylallyl pyrophosphate synthase (Aa409), β-amyrin synthase (Aa401), amorpha-4,11-diene synthase (Aa408), *epi*-cedrol synthase (Aa413), β-caryophyllene synthase (Aa417), (*3R*)-linalool synthase (Aa442, Aa443), β-pinene synthase (Aa456), amorpha-4,11-diene C-12 oxidase (Aa557, Aa561), steroid 23-α-hydroxylase (Aa21), taxadiene 5-α-hydroxylase (Aa25), *etc*. Interestingly, some genes like sesquiterpene cyclase were found to be present in both categories – up-regulated in seedling (Aa555) and down-regulated in seedling (Aa417), which may be due to presence of different isoforms. In addition, the transcript abundance of *HMGR* (3-hydroxy-3-methyl-glutaryl-CoA reductase) (Aa689) was observed to be lower in the seedlings (fold change 0.5), whereas the transcript abundance of *DXR* (1-deoxy-D-xylulose 5-phosphate reductoisomerase) (Aa554) was higher in the seedlings compared to the mature leaf (fold change 2.55). This possibly indicates that the plastidial non-mevalonate terpenoid pathway contributing the major metabolic flux for isopentenyl pyrophosphate (IPP) biosynthesis at the seedling stage, whereas the cytosolic mevalonate pathway is the more prominent flux contributor in the leaf at mature stage. This is consistent with the fact that the artemisinin content of the mature leaf in *A. annua* is much higher than that in the seedling, and artemisinin being a sesquiterpene (C_15_) is produced in the cytosolic compartment of the plant cell with major metabolic flux for the intermediate IPP coming from the mevalonate pathway. However, this hypothesis needs further validation as interestingly, 1-deoxy-D-xylulose-5-phosphate synthase (*DXS*, Aa459) and 2-*C*-methyl-D-erythritol 2,4-cyclodiphosphate synthase (*MECS*; Aa430) of the non-mevalonate pathway showed higher transcript abundance in the mature leaf whereas an *HMGR* isoform (Aa308) showed higher transcript abundance in seedling. This may be due to differential expression of isoforms in different tissue. But, as reported earlier the non-mevalonate pathway predominates over the mevalonate pathway in the *A. annua* GTs [34] and is another reason to analyze the function of these isoforms in detail.

Among the selections, a few interesting candidates were observed with the potential for functional characterization. For example, a few genes (like Aa745) showing no significant similarity to database sequences could provide further clues related to artemisinin biosynthesis and/or accumulation in the plant. Among the in-house *cyp* genes on the array, only Aa590 was found to have higher transcript abundance in the seedling as compared to the mature leaf. Another interesting class of differentially expressing genes belonged to the category – peroxidases. It has been shown earlier in *Arabidopsis* that from seedling to mature plant, in cotyledons or leaves of different ages, plastidial gene expression is regulated at the transcriptional and post-transcriptional levels, but not by plastome copy number [35]. This emphasises the role of transcriptional regulators in the transition from seedling to mature leaf in a plant and must be true for non-plastidial genes too. This view was reinforced by the identification of several differentially expressing transcription factors in the *A. annua* mature leaf *vis-à-vis* seedling. For example, nuclear transcription factor Y subunit C-1 (Aa94), AP2/ERF and B3 domain-containing transcription factor RAV1(Aa127), transcription factor AS1 (Aa185), ethylene-responsive transcription factor ERF025 (Aa216), *etc* were found to be having higher expression in seedling compared to mature leaf. On the other hand, multi-protein bridging factor 1A (Aa91), homeobox protein knotted-1-like 7 (Aa111), NAC domain containing protein 83 (Aa138, Aa244), DELLA protein RGA (Aa141), BEL1-like homeodomain 1 (Aa143), auxin-responsive protein IAA9 (Aa163), transcription factor ILR3 (Aa167), RNA polymerase sigma factor (Aa171), transcription factor TCP7 (Aa206), basic leucine-zipper 44 (Aa226), protein agamous-like 42 (Aa233), AP2 domain transcriptional regulator (Aa241), nuclear factor Y (Aa247), scarecrow-like protein 30 (Aa266), homeodomain-like transcriptional regulator (Aa271), *etc* were found to show higher transcript abundance in mature leaf as compared to seedling.

As there is a possible linkage between flowering and artemisinin biosynthesis [36], the genes associated with flowering with higher transcript abundance in mature leaf assume significance. BELL (BEL1) is known to control ovule development through negative regulation of AGAMOUS gene (AG) in *Arabidopsis* [37] and the DELLA protein is a target for gibberellin signalling [38]. Gibberellins are known to be enhancing flowering in plants. As the artemisinin content in the leaf peaks just before flowering, it is easy to link the flowering regulators with the role to enhance artemisinin content directly or indirectly. But, higher expression of the early flowering gene CONSTANS in mature leaf compared to seedling, did not induce an increase in artemisinin biosynthesis [39]. Taken together, these results suggest that the observed increase of artemisinin content at pre-flowering stage may not be a direct consequence of flowering itself, but may be due to the combined influence of factors preparing the plants to proceed for flowering stage.

### Conclusion

Selected genes from the EST database and in-house generated ESTs from trichomes were analyzed for differential expression at contrasting stages. This is the first report on microarray analysis relating the expression to differential stages of artemisinin biosynthesis. More number of genes were found to show higher transcript abundance in the leaf as compared to seedling indicating a higher level of the metabolic complexity of the mature leaf *vis-à-vis* the seedling. The result was validated by quantitative and semiquantitative RT-PCR for known genes and found to be confirming the expression of metabolites and result of microarray experiment. Several transcriptional regulators were indicated for their higher expression in the mature leaf and their functional characterization will provide further insight into artemisinin biosynthesis. Higher expression of genes associated with flowering in mature leaf indicates the preparation for flowering, which may be also indirectly influencing artemisinin biosynthesis.

## Supporting Information

Figure S1 **Heat map for the differentially expressing genes upregulated in seedling.**
(TIF)

Figure S2 **Heat map for the differentially expressing genes downregulated in seedling.**
(TIF)

Table S1 **Details of in-house cytochrome P450 sequences of** ***A. annua*** **.**
(DOC)

Table S2 **Sequences of the primers used for semi-quantitative RT-PCR-based expression analysis of selected target genes of** ***A. annua*** **.**
(DOC)

Table S3 **Sequence details of Assay-By-Designs for TaqMan chemistry-based Real Time PCR of** ***A. annua*** **sesquiterpene biosynthetic pathway genes.**
(DOC)

Table S4 **Genes found to be down-regulated in seedling as compared to mature plant leaf of** ***A. annua*** **.**
(DOC)

Table S5 **Genes found to be up-regulated in seedling as compared to mature plant leaf in** ***A. annua*** **.**
(DOC)
