# Supplementary figures and images for: Differentially Expressed Genes during Contrasting Growth Stages of Artemisia annua for Artemisinin Content
Source: PLoS One. 2013 Apr 3;8(4):e60375. doi: 10.1371/journal.pone.0060375 (PMC3616052; doi:10.1371/journal.pone.0060375)

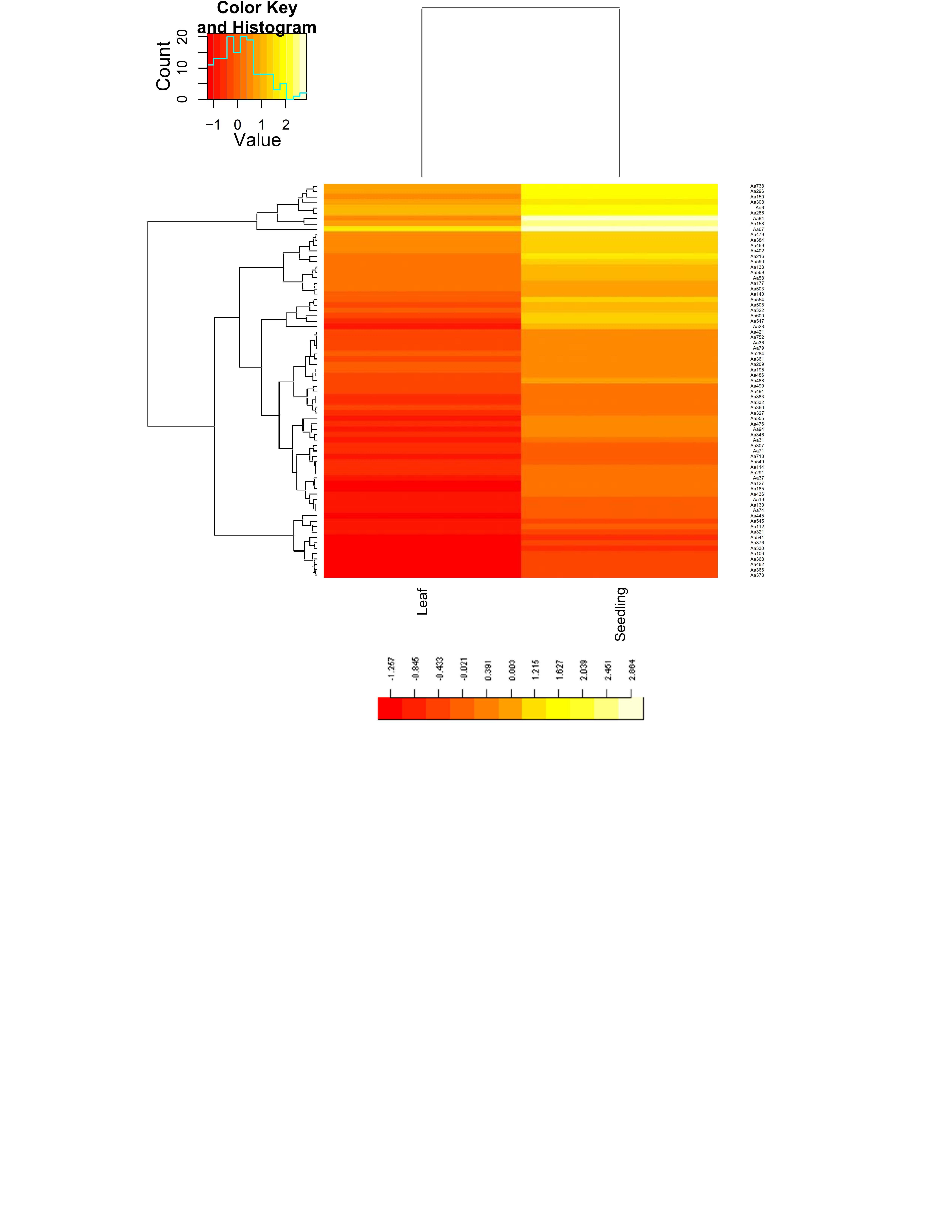

Supplement: Figure S1 — Heat map for the differentially expressing genes upregulated in seedling. (TIF) [file pone.0060375.s001.tif]

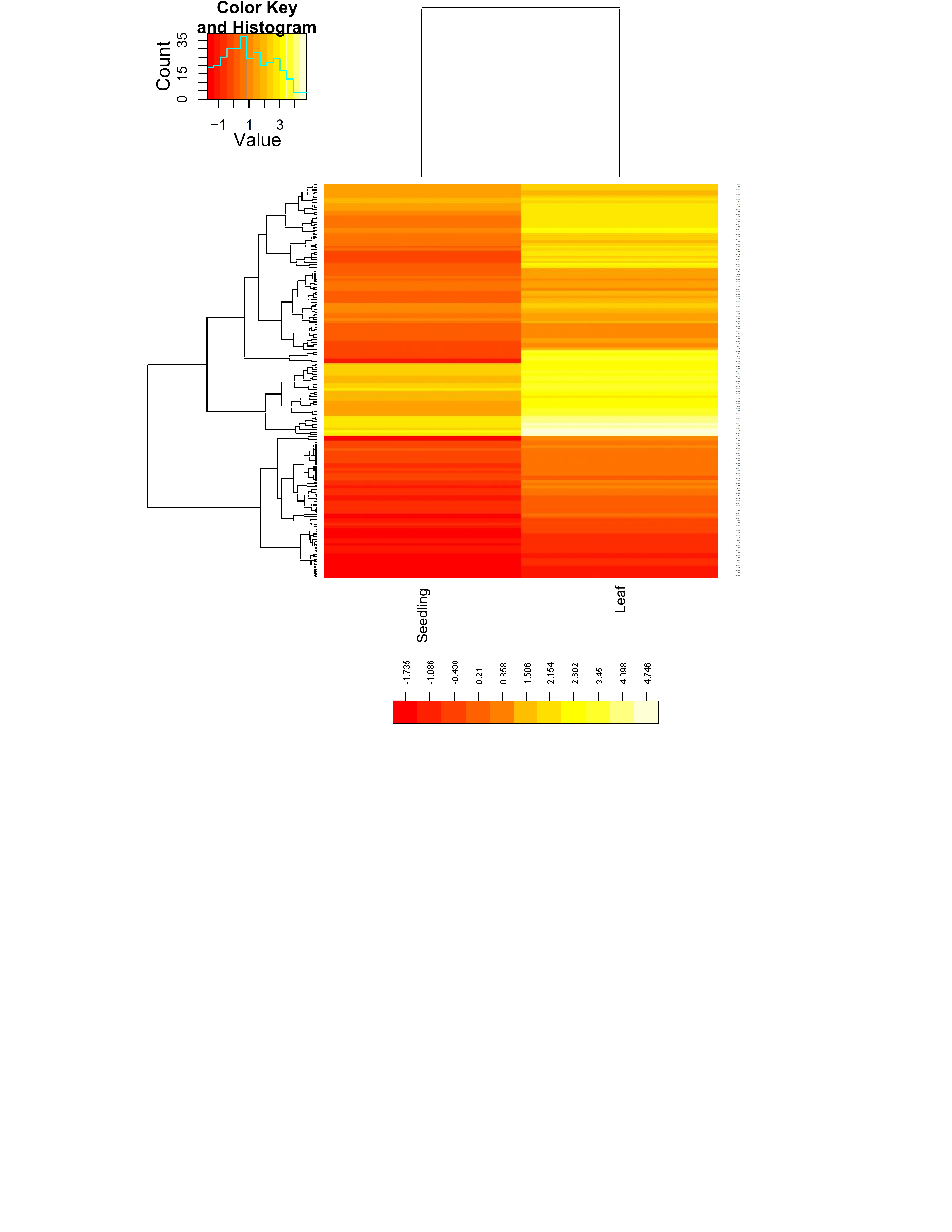

Supplement: Figure S2 — Heat map for the differentially expressing genes downregulated in seedling. (TIF) [file pone.0060375.s002.tif]
