# Supplementary material for: Differentially Expressed Genes during Contrasting Growth Stages of Artemisia annua for Artemisinin Content
Source: PLoS One. 2013 Apr 3;8(4):e60375. doi: 10.1371/journal.pone.0060375 (PMC3616052; doi:10.1371/journal.pone.0060375)
Supplement: Table S1 — Details of in-house cytochrome P450 sequences of A. annua . (DOC) [file pone.0060375.s003.doc]

**Table S1.** Details of in-house cytochrome P450 sequences of *A. annua*.

| **S. No in MA** | **Sequence Name** | **GenBank accession no.** |
| --- | --- | --- |
| Aa0020 | CIM-Arog_CYP01_1533 | KC594703 |
| Aa0576 | CIM-Arog_CYP02_1518 | GU318226 |
| Aa0577 | CIM-Arog_CYP03_1551 | GU318229 |
| Aa0032 | CIM-Arog_CYP04_1548 | JN594505 |
| Not spotted | CIM-Arog_CYP05_1509 | JN594506 |
| Aa0062 | CIM-Arog_CYP06_1476 | GU318228 |
| Aa0578 | CIM-Arog_CYP07_1542 | GU318230 |
| Aa0062 | CIM-Arog_CYP08_1572 | KC594704 |
| Aa0579 | CIM-Arog_CYP09_537 | GU318242 |
| Aa0580 | CIM-Arog_CYP10_377 | GU318240 |
| Aa0581 | CIM-Arog_CYP11_263 | GU318239 |
| Aa0582 | CIM-Arog_CYP12_497 | GU318237 |
| Aa0583 | CIM-Arog_CYP13_388 | GU318241 |
| Aa0584 | CIM-Arog_CYP14_398 | GU318238 |
| Aa0585 | CIM-Arog_CYP15_417 | GU318236 |
| Aa0586 | CIM-Arog_CYP16_434 | GU318235 |
| Aa0587 | CIM-Arog_CYP17_368 | GU318234 |
| Aa0588 | CIM-Arog_CYP18_286 | GU318243 |
| Aa0589 | CIM-Arog_CYP19_486 | KC594705 |
| Aa0590 | CIM-Arog_CYP20_333 | GU318232 |
| Aa0591 | CIM-Arog_CYP21_387 | GU318233 |
| Aa0592 | CIM-Arog_CYP22_521 | GU318231 |
