# Supplementary material for: Differentially Expressed Genes during Contrasting Growth Stages of Artemisia annua for Artemisinin Content
Source: PLoS One. 2013 Apr 3;8(4):e60375. doi: 10.1371/journal.pone.0060375 (PMC3616052; doi:10.1371/journal.pone.0060375)
Supplement: Table S2 — Sequences of the primers used for semi-quantitative RT-PCR-based expression analysis of selected target genes of A. annua . (DOC) [file pone.0060375.s004.doc]

**Table S2:** Sequences of the primers used for semi-quantitative RT-PCR-based expression analysis of selected target genes of *A. annua*.

| **S. No.** | **Gene name** | **Primer sequence**  **(5’-3’)** | |
| --- | --- | --- | --- |
| 1 | Aa91 | F | GCTCCAACTTCCGCCGCTCGTAAGG |
|  |  | R | GGGCCCTTTCCAGTTTGGTGATGATC |
| 2 | Aa316 | F | ATGTCTGAAAAACCAACCTTG |
|  |  | R | CTAGAGGCGTGACAGTCGTTCAAC |
| 3 | Aa335 | F | GCAGGAACCACGGCTCTTGTAACTG |
|  |  | R | TCTTTGGCCCATTCACATGCTAAATTTC |
| 4 | Aa340 | F | CTTTGCTGCTGATCCTGACTTCCTAGAG |
|  |  | R | CAATCGAAGTCTTCACAGCATCCAACAC |
| 5 | Aa347 | F | TCTCCTCCTGATCAGAAGATCCCTTTTC |
|  |  | R | CCTTTCTTCAGCTACCGCCTTGTCTTTG |
| 6 | Aa372 | F | CTTCAGGTGTGGCACTTGTCTTGATTGG |
|  |  | R | CCTGCTTCTTTGTCCCTGTGGATGTATG |
| 7 | Aa379 | F | CCCCGGTATGTTCGCCTCGATCAAGATC |
|  |  | R | CTTCTAGGGTGTCTCTGAATGGCAAAGG |
| 8 | Aa388 | F | GTTGGTGGGTGGAACTCGAGCAACAC |
|  |  | R | TGCAGGGCTTCTTCACGTTTTATCTC |
| 9 | Aa393 | F | GGCCGCCGACTTTGCTTGATTTTGA |
|  |  | R | CTGAGAACCCCATCCAATAACACCAATC |
| 10 | Aa396 | F | CATCCCCCATTTCTCTAACCGCTCTAC |
|  |  | R | GCTTCCGCTGCAGGTACTTCAGTTTG |
| 11 | Aa409 | F | ATGGCGTCTTTGCAGCTAACACCTC |
|  |  | R | CTACACCAATTGCAGGGCCTCCTC |
| 12 | Aa415 | F | ATCGGGGCAGGAGGATTTATCGGATCAC |
|  |  | R | TGAGCCAAAAATGCAAGGGGAAGTATC |
| 13 | Aa446 | F | GTGATCGATCTTAGCCAGACGGATAACG |
|  |  | R | CTCGGGGCATGGACATGGTGGGTAGTAG |
| 14 | Aa525 | F | TTGATACCATGGGAAAGTGCTAC |
|  |  | R | TTAGGCCTCAGCAAATCTCAATTC |
| 15 | Aa528 | F | GCTGTTGAGAGAGAGTGTCCAGGAGTTG |
|  |  | R | AGTTTCATAGGGGTGCCACGGTCATTTC |
| 16 | Aa530 | F | CCATGATTGTTTCGTCAGGGGTTGTG |
|  |  | R | GAAGCAATTGATACCGTCCCATCTCTTC |
| 17 | Aa540 | F | CCTGGGCTATCGTACACATTCTACCGG |
|  |  | R | AAGAAACTGTTCGGTTGCAAGCGTTGTG |
| 18 | Aa542 | F | CTGCACCAATACAACCACTCCTCAAGAG |
|  |  | R | AAGGCGCTTCTTGAAGACTCTGTATTCC |
| 19 | Aa547 | F | GGGTCGCAGAGATGGGTTAGTTTCACG |
|  |  | R | GGAAAGATGCGTCAATAGTCGGGTCAGG |
| 20 | Aa548 | F | GGAGACAACAATTTGGCACCGCTAGATG |
|  |  | R | ATCTCACCACTTGATCCAGTTAGAGGCC |
| 21 | Aa555 | F | ATGTCGACTCTTCCTATTTCTAGTG |
|  |  | R | TTAGACAACCATAGGGTGAACGAAG |
| 22 | Aa556 | F | ATGGAAGACTGTGATGTACACAACCTGC |
|  |  | R | TTAAGCACTAAAGCCGTTGTAAATAGAG |
| 23 | Aa627 | F | GGGCGATCGGAGAGATTGGTGGTGGAAG |
|  |  | R | GGCCAATGTCTATGATAAAAACTCTCGC |
| 24 | Aa635 | F | ACAAAGCAGCACAAGTCAAGGAAGCACC |
|  |  | R | CAAAACAACTCAGCAGGTGGAGGCAGG |
| 25 | Aa658 | F | GCATCCCATATCACAACAAAGTGCTTC |
|  |  | R | CCACCCTCCACCACCTGTTGTCCAC |
| 26 | Aa677 | F | TCGAGTTGGTTTAGTTTAGCATCCCGTC |
|  |  | R | GACCCGAGATTGTGATCACACCTGAAGG |
| 27 | Aa691 | F | CCTCTCGAGGCCCTTGGGAGCGTGTC |
|  |  | R | CAAAATAGTTTCGGCCTCTTTCCCATGG |
| 28 | Aa694 | F | TGTGGTATACGTACTGGATTGGCGAGCC |
|  |  | R | AACATCACTGTCTTTTAGAGTCGGGGGG |
| 29 | Aa701 | F | CGGTGGTCGTCGTGGTGTTGTGAACTCG |
|  |  | R | GGGTTCAAACCTCGTGGATTAGCCGG |
| 30 | Aa702 | F | GGGTGCACGTCGTCGTCCAGTTGGCAAG |
|  |  | R | CGGTGAGTTGATGATGCCATAAGAGAGG |
| 31 | Aa709 | F | GTCGACCGTGAGGGAAATGTTGTTGATC |
|  |  | R | CGTGACCGGCAACAAAACTAGATTAAAG |
| 32 | Aa731 | F | CATTGCCACATTGAGCCTCATTTGCAC |
|  |  | R | ATTTTAAGGGGCCGATATACACGACGC |
| 33 | Aa738 | F | CTTGCCAACTTCACTCGTGAGAGGATCC |
|  |  | R | ACTTCGGGGAACGCCATTGCATCACGAG |
| 34 | Aa741 | F | GAGGAGAGAAGTCGCCATTGGCTATGTG |
|  |  | R | GTAAAACAAGAGGCACCATGCAGGGAC |
| 35 | Aa742 | F | GTGAAACCATTTATGGGCTTGTGCAGTG |
|  |  | R | CCTGGTGATGGCGGCGAAACTTGC |
| 36 | Aa745 | F | GCGGAAGTTGGTGATGTCAATCTCGACG |
|  |  | R | GACTTCCTGATTCCACCTGTGGTTCGAG |
| 37 | Aa actin  (Based on EU531837) | F | AGCAACTGGGATGACATGGAGAAG |
|  |  | R | ATCTTCATGCTGCTCGGGGC |
