# Supplementary material for: Differentially Expressed Genes during Contrasting Growth Stages of Artemisia annua for Artemisinin Content
Source: PLoS One. 2013 Apr 3;8(4):e60375. doi: 10.1371/journal.pone.0060375 (PMC3616052; doi:10.1371/journal.pone.0060375)
Supplement: Table S3 — Sequence details of Assay-By-Designs for TaqMan chemistry-based Real Time PCR of A. annua sesquiterpene biosynthetic pathway genes. (DOC) [file pone.0060375.s005.doc]

| **S. No.** | **Gene ID** | **Gene** | **Forward Primer Sequence** | **Reverse Primer Sequence** | **Reporter Sequence** |
| --- | --- | --- | --- | --- | --- |
| 1 | Aa398 | Farnesyl pyrophosphate synthase | CACTACACTTGTTGGAGAGAAAGATCTC | AGGCAACTGGAAGGTAAAATGAGTAG | CACCGCCGAATTGT |
| 2 | Aa15 | Cytochrome P450 reductase (CPR) | GATGTTGTGGACGAAGCTGAAAA | CGTCTTCGTTATCAGCGTGTACTG | CCGCCGCACACTTA |
| 3 | Aa408 | Amorphadiene synthase | CCCGGGCTAGAGTTTTCTTCA | GTACCATACGCATCATAAGTGTCATCT | ACAGCAACAGCTTTTG |
| 4 | Aa561 | P450 monooxygenase (CYP71) | GTGGCAGACATCTTTCCTTCGA | TCGAGCTTCATGTGAATGCTAGTT | CCGAGCTCTCTTGCCC |
| 5 | Aa442 | Linalool Synthase | CCAAACTTTAGTCATGGGAGGAGAA | GCCCAAAGTACCAAACACATCATAG | TCATAGCGGCAACCTT |
| 6 | Aa434 | Germacrene-A synthase | GGCTTTAGTTGGTATGGGTGAGATT | ACTCAGAAGCCTGCAAAGTCTTT | AAGCCAAAGCATCTTC |
| 7 | Aa407 | Squalene synthase | TCGAGGATATTAATGAGATACCTAAGTCACG | CTTCATATTTCAGGTCCTCTAGTTTATTCACA | ACGAGGCCAAAACAT |
| 8 | Aa417 | β –Caryophyllene synthase | CAATCATTGCATAGAAAGGAGCTTAGC | AGGTAGATTGTTTGGGACATCAAGAC | CCACCACCTGGAAACT |
| 9 | Aa560 | Sesquiterpene cyclase | TGCTTCAATCAATACACAAAAGGGAGAT | GCGGTAGTTTGTTTGAGAAATCCAA | ACCATTTGCAGATTTC |
| 10 | Aa554 | 1-Deoxy-D-xylulose-5-phosphate reductoisomerase (DXR) | CTGGCGGTCCCTTTGTTCT | TGAATCAGCAGGAAGAATCTTGACAT | CTTGCGCACAAACAT |
| 11 | Aa689 | HMG-CoA reductase | GCGCCTGTTGTTAGGTTTGC | TGTCAAAATTCACCCCATCTTCCAA | CTGCGAAGCCCTCTCC |

**Table S3:** Sequence details of Assay-By-Designs for TaqMan chemistry-based Real Time PCR of *A. annua* sesquiterpene biosynthetic pathway genes.
