# Supplementary material for: Differentially Expressed Genes during Contrasting Growth Stages of Artemisia annua for Artemisinin Content
Source: PLoS One. 2013 Apr 3;8(4):e60375. doi: 10.1371/journal.pone.0060375 (PMC3616052; doi:10.1371/journal.pone.0060375)
Supplement: Table S4 — Genes found to be down-regulated in seedling as compared to mature plant leaf of A. annua . (DOC) [file pone.0060375.s006.doc]

**Table S4:** Genes found to be down-regulated in seedling as compared to mature plant leaf of *Artemisia annua*.

| **S. No.** | **Gene / Probe ID** | **Gene / Probe Homology-based Annotation / Description** | **Log Fold Change** | **Fold Change** |
| --- | --- | --- | --- | --- |
|  | Aa1 | Cytochrome b,Ubiquinol-cytochrome-c reductase complex cytochrome b subunit ( P42792) [*Arabidopsis thaliana*] | -0.665517 | 0.630463 |
|  | Aa3 | cytochrome c oxidase subunit 1 [*Arabidopsis thaliana*] (NP_085587) | -0.654993 | 0.635078 |
|  | Aa15 | *Artemisia annua* cytochrome P450 reductase (EF197890) | -1.378634 | 0.384583 |
|  | Aa17 | cytochrome c biogenesis protein [*Arabidopsis thaliana*] (NP_051108) | -0.607105 | 0.656513 |
|  | Aa21 | *Artemisia annua* putative steroid 23-alpha-hydroxylase cytochrome P450 mRNA(DQ363132) | -0.978760 | 0.507416 |
|  | Aa25 | *Artemisia annua* putative taxadiene 5-alpha-hydroxylase cytochrome P450 mRNA, (DQ363134) | -3.832573 | 0.070191 |
|  | Aa26 | cytochrome b5 isoform 1 [*Arabidopsis thaliana*] (NP_200168) | -1.554010 | 0.340562 |
|  | Aa27 | cytochrome b5 isoform 1 [*Arabidopsis thaliana*] (NP_200168) | -2.297156 | 0.203464 |
|  | Aa29 | abscisic acid 8'-hydroxylase 2 [*Arabidopsis thaliana*] (NP_180473) | -1.358201 | 0.390068 |
|  | Aa30 | cytochrome b5 isoform 1 [*Arabidopsis thaliana*] (NP_200168) | -0.932884 | 0.523810 |
|  | Aa32 | cytochrome P450, family 72, subfamily A, polypeptide 15 [*Arabidopsis thaliana*] (NP_188087) | -1.072802 | 0.475395 |
|  | Aa33 | cytochrome b561 [*Arabidopsis thaliana*] (AAM62824) | -1.543080 | 0.343152 |
|  | Aa51 | *Artemisia annua* putative flavonoid 3 -hydroxylase cytochrome P450 mRNA, (DQ363131) | -1.501530 | 0.353179 |
|  | Aa53 | *Artemisia annua* putative taxane 13-alpha-hydroxylase cytochrome P450 mRNA, (DQ363133) | -0.840187 | 0.558571 |
|  | Aa62 | cytochrome P450, family 82, subfamily C, polypeptide 4 [*Arabidopsis thaliana*] (NP_194922) | -0.851449 | 0.554228 |
|  | Aa68 | cytochrome P450, family 93, subfamily D, polypeptide 1 [*Arabidopsis thaliana*] (NP_196307) | -0.584309 | 0.666969 |
|  | Aa85 | cytochrome P450, family 715, subfamily A, polypeptide 1 [*Arabidopsis thaliana*] (NP_200053) | -0.896681 | 0.537121 |
|  | Aa86 | cytochrome P450 71A21 [*Arabidopsis thaliana*] (NP_680111) | -0.602189 | 0.658753 |
|  | Aa91 | multiprotein bridging factor 1A [*Arabidopsis thaliana*] (NP_565981) | -1.652788 | 0.318025 |
|  | Aa92 | nuclear transcription factor Y subunit B-3 [*Arabidopsis thaliana*] (NP_193190) | -0.860623 | 0.550715 |
|  | Aa93 | Similar to Schizosaccharomyces CCAAT-binding factor. [*Arabidopsis thaliana*] (AAB70410) | -1.211259 | 0.431892 |
|  | Aa95 | histone deacetylase complex subunit SAP18 [*Arabidopsis thaliana*] (NP_566050) | -0.834304 | 0.560853 |
|  | Aa108 | ureidoglycine aminohydrolase [*Arabidopsis thaliana*] (NP_193438) | -0.600532 | 0.659511 |
|  | Aa111 | homeobox protein knotted-1-like 7 [*Arabidopsis thaliana*] (NP_564805) | -1.488941 | 0.356274 |
|  | Aa122 | Tubby-like F-box protein 7 [*Arabidopsis thaliana*] (NP_564627) | -0.683552 | 0.622630 |
|  | Aa138 | NAC domain containing protein 83 [*Arabidopsis thaliana*] (NP_196822) | -1.225093 | 0.427770 |
|  | Aa141 | DELLA protein RGA [*Arabidopsis thaliana*] (NP_178266) | -0.952092 | 0.516882 |
|  | Aa143 | BEL1-like homeodomain 1 [*Arabidopsis thaliana*] (AAK43836) | -2.702299 | 0.153648 |
|  | Aa144 | plastid transcriptionally active 4 [*Arabidopsis thaliana*] (NP_564846) | -0.813577 | 0.568969 |
|  | Aa151 | BEL1-like homeodomain 1 [*Arabidopsis thaliana*] (NP_181138) | -0.699182 | 0.615922 |
|  | Aa153 | Salt tolerance protein [*Arabidopsis thaliana*] (NP_172094) | -0.827561 | 0.563481 |
|  | Aa163 | auxin-responsive protein IAA9 [*Arabidopsis thaliana*] (NP_569017) | -1.336100 | 0.396090 |
|  | Aa167 | transcription factor ILR3 [*Arabidopsis thaliana*] (NP_200279) | -1.033723 | 0.488448 |
|  | Aa169 | B3 domain-containing transcription repressor VAL2 [*Arabidopsis thaliana*] (NP_194929) | -0.693519 | 0.618344 |
|  | Aa170 | transcription elongation factor SPT6 [*Arabidopsis thaliana*] (NP_001185317) | -0.675925 | 0.625931 |
|  | Aa171 | RNA polymerase sigma factor [*Arabidopsis thaliana*] (NP_197800) | -3.680140 | 0.078013 |
|  | Aa174 | protein ethylene insensitive 3 [*Arabidopsis thaliana*] (NP_188713) | -0.663152 | 0.631497 |
|  | Aa186 | uncharacterized protein [*Arabidopsis thaliana*] (NP_173030) | -0.934318 | 0.523290 |
|  | Aa193 | WRKY transcription factor 1 splice variant 1 [*Arabidopsis thaliana*] (BAE98477) | -0.858884 | 0.551379 |
|  | Aa196 | transcription factor BPE [*Arabidopsis thaliana*] (NP_564749) | -0.787675 | 0.579277 |
|  | Aa198 | ethylene-responsive transcription factor 1A [*Arabidopsis thaliana*] (NP_567530) | -0.883567 | 0.542026 |
|  | Aa206 | transcription factor TCP7 [*Arabidopsis thaliana*] (NP_197719) | -1.629547 | 0.323190 |
|  | Aa207 | indole-3-acetic acid inducible 4 [*Arabidopsis thaliana*] (ADC29372) | -1.078584 | 0.473493 |
|  | Aa220 | putative WRKY transcription factor 15 [*Arabidopsis thaliana*] (NP_179913) | -0.652065 | 0.636369 |
|  | Aa226 | basic leucine-zipper 44 [*Arabidopsis thaliana*] (NP_177672) | -1.262889 | 0.416709 |
|  | Aa231 | auxin response factor 9 [*Arabidopsis thaliana*] (NP_194129) | -0.733853 | 0.601296 |
|  | Aa233 | protein agamous-like 42 [*Arabidopsis thaliana*] (NP_568952) | -2.832735 | 0.140366 |
|  | Aa241 | putative AP2 domain transcriptional regulator, 5' partial; 1-558 [*Arabidopsis thaliana*] (AAG52091) | -2.208228 | 0.216400 |
|  | Aa244 | NAC domain containing protein 83 [*Arabidopsis thaliana*] (NP_196822) | -1.485032 | 0.357241 |
|  | Aa247 | nuclear factor Y, subunit C13 [*Arabidopsis thaliana*] (NP_199139) | -1.266693 | 0.415611 |
|  | Aa254 | RNA polymerase II transcriptional coactivator KIWI [*Arabidopsis thaliana*] (NP_196487) | -0.881497 | 0.542804 |
|  | Aa259 | protein ethylene insensitive 3 [*Arabidopsis thaliana*] (NP_188713) | -0.654678 | 0.635217 |
|  | Aa264 | scarecrow-like protein 15 [*Arabidopsis thaliana*] (NP_195389) | -0.667750 | 0.629488 |
|  | Aa266 | scarecrow-like protein 30 [*Arabidopsis thaliana*] (NP_001078251) | -1.359046 | 0.389840 |
|  | Aa268 | two-component response regulator-like APRR3 [*Arabidopsis thaliana*] (NP_001190575) | -1.127476 | 0.457716 |
|  | Aa269 | WRKY transcription factor 54 [*Arabidopsis thaliana*] (AAL29430) | -0.738033 | 0.599556 |
|  | Aa271 | Homeodomain-like transcriptional regulator [*Arabidopsis thaliana*] (NP_001190470) | -1.390764 | 0.381363 |
|  | Aa274 | sequence-specific DNA binding transcription factor [*Arabidopsis thaliana*] (NP_194855) | -0.764182 | 0.588787 |
|  | Aa275 | two-component response regulator-like APRR5 [*Arabidopsis thaliana*] (Q6LA42) | -1.223519 | 0.428237 |
|  | Aa277 | putative WRKY DNA-binding protein 7 [*Arabidopsis thaliana*] (NP_194155) | -0.608487 | 0.655884 |
|  | Aa285 | phosphate transporter [*Arabidopsis thaliana*] (BAE98881) | -3.114600 | 0.115455 |
|  | Aa289 | putative Mn-specific cation diffusion facilitator transporter [*Arabidopsis thaliana*] (NP_181477) | -2.310703 | 0.201562 |
|  | Aa301 | Clathrin light chain protein [*Arabidopsis thaliana*] (NP_565484) | -0.795536 | 0.576129 |
|  | Aa316 | *Artemisia annua* voucher huayang2 12-oxophytodienoate reductase-like protein mRNA, (EU848577)/ *Artemisia annua* artemisinic aldehyde delta-11(13) reductase (ACH61780.1) | -1.006099 | 0.497891 |
|  | Aa317 | NADPH-ferrihemoprotein reductase [*Arabidopsis thaliana*] (NP_194183) | -0.611520 | 0.654507 |
|  | Aa334 | Rossmann-fold NAD(P)-binding domain-containing protein [*Arabidopsis thaliana*] (NP_194073) | -0.626339 | 0.647818 |
|  | Aa335 | tropine dehydrogenase [*Arabidopsis thaliana*] (NP_196225) | -1.516377 | 0.349563 |
|  | Aa340 | putative AX110P protein [*Arabidopsis thaliana*] (AAK76525) | -1.328382 | 0.398215 |
|  | Aa347 | clavaminate synthase-like protein [*Arabidopsis thaliana*] NAD(P)-linked oxidoreductase-like protein [*Arabidopsis thaliana*] (NP_188773) | -1.137866 | 0.454431 |
|  | Aa350 | NAD(P)-linked oxidoreductase-like protein [*Arabidopsis thaliana*] (NP_564761) | -0.768031 | 0.587218 |
|  | Aa356 | Rossmann-fold NAD(P)-binding domain-containing protein [*Arabidopsis thaliana*] (NP_563866) | -0.722698 | 0.605963 |
|  | Aa372 | Thioredoxin-like protein [*Arabidopsis thaliana*] (NP_030274) | -2.248172 | 0.210491 |
|  | Aa379 | protein SRG1 [*Arabidopsis thaliana*] (NP_173145) | -1.486589 | 0.356855 |
|  | Aa388 | *Artemisia annua*  IPP/DMAPP synthase (ABY57296.1) | -1.831610 | 0.280951 |
|  | Aa393 | ketol-acid reductoisomerase [*Arabidopsis thaliana*] (AAW82381) | -1.348233 | 0.392773 |
|  | Aa395 | 4-hydroxy-3-methylbut-2-enyl diphosphate reductase [*Arabidopsis thaliana*] (NP_567965) | -0.851572 | 0.554180 |
|  | Aa396 | male sterility 2-like protein [*Arabidopsis thaliana*] (CAA20592) | -1.075108 | 0.474636 |
|  | Aa401 | *Artemisia annua* beta-amyrin synthase (BAS) mRNA, complete cds (EU563939) | -0.991458 | 0.502969 |
|  | Aa407 | *Artemisia annua* squalene synthase mRNA, complete cds (AF302464) | -1.104670 | 0.465009 |
|  | Aa408 | *Artemisia annua* mRNA for amorpha-4,11-diene synthase (kcs12 gene) (AJ251751) | -2.108200 | 0.231936 |
|  | Aa409 | *Artemisia annua* IPP/DMAPP synthase (ispH) mRNA, complete cds (EU332141) | -2.447293 | 0.183354 |
|  | Aa413 | *Artemisia annua* 8-epicedrol synthase (Ecs1) mRNA, complete cds (AF157059) | -1.725299 | 0.302436 |
|  | Aa415 | similar to dihydroflavonol reductase [*Arabidopsis thaliana*] (AAK68820) | -1.413989 | 0.375273 |
|  | Aa417 | *Artemisia annua* mRNA for putative sesquiterpene cyclase (cASC34 gene) (AJ271793) | -1.087717 | 0.470505 |
|  | Aa430 | 2C-methyl-D-erythritol 2,4-cyclodiphosphate synthase [*Arabidopsis thaliana*] (AAM62786) | -1.230706 | 0.426109 |
|  | Aa432 | mutant protein of chalcone synthase [*Arabidopsis thaliana*] (BAD89854) | -0.957990 | 0.514773 |
|  | Aa434 | *Artemisia annua* germacrene A synthase (GAS) mRNA, complete cds (DQ447636) | -0.834318 | 0.560848 |
|  | Aa442 | *Artemisia annua* (3R)-linalool synthase (QH5) mRNA, complete cds (AF154124) | -2.933513 | 0.130895 |
|  | Aa443 | *Artemisia annua* (3R)-linalool synthase (QH1) mRNA,complete cds (AF154125) | -1.907274 | 0.266596 |
|  | Aa446 | flavone synthase [*Arabidopsis thaliana*] (CAP09039) | -1.636486 | 0.321639 |
|  | Aa456 | *Artemisia annua* (-)-beta-pinene synthase (QH6) mRNA, complete cds (AF276072) | -1.201715 | 0.434758 |
|  | Aa458 | putative long-chain-alcohol O-fatty-acyltransferase 8 [*Arabidopsis thaliana*] (NP_174711) | -0.716200 | 0.608699 |
|  | Aa459 | *Artemisia annua* 1-deoxy-D-xylulose 5-phosphate synthase 3 (AAD56390.2) | -0.796371 | 0.575796 |
|  | Aa481 | 2-oxoisovalerate dehydrogenase E1 component, alpha subunit [*Arabidopsis thaliana*] (NP_974756) | -0.709215 | 0.611653 |
|  | Aa483 | 3-hydroxyisobutyrate dehydrogenase [*Arabidopsis thaliana*] (NP_567617) | -1.020856 | 0.492824 |
|  | Aa497 | 3-hydroxybutyryl-CoA dehydrogenase [*Arabidopsis thaliana*] (NP_188147) | -0.834519 | 0.560770 |
|  | Aa518 | GSH-dependent dehydroascorbate reductase 1, putative [*Arabidopsis thaliana*] (AAM65005) | -0.805415 | 0.572197 |
|  | Aa520 | mitochondrial aldehyde dehydrogenase [*Arabidopsis thaliana*] (AAL99612) | -0.840492 | 0.558453 |
|  | Aa523 | 2-cys peroxiredoxin-like protein [*Arabidopsis thaliana*] (AAM62760) | -0.790773 | 0.578034 |
|  | Aa525 | L-ascorbate peroxidase [*Arabidopsis thaliana*] (NP_172267) | -1.787790 | 0.289615 |
|  | Aa526 | L-ascorbate peroxidase [*Arabidopsis thaliana*] (NP_195226) | -0.909089 | 0.532521 |
|  | Aa528 | peroxidase ATP1a [*Arabidopsis thaliana*] (CAA66862) | -1.049413 | 0.483165 |
|  | Aa530 | peroxidase 3 [*Arabidopsis thaliana*] (NP_172018) | -2.260808 | 0.208655 |
|  | Aa534 | peroxidase 39 [*Arabidopsis thaliana*] (NP_192868) | -0.649719 | 0.637404 |
|  | Aa540 | peroxidase 12 [*Arabidopsis thaliana*] (NP_177313) | -1.601021 | 0.329644 |
|  | Aa542 | L-ascorbate peroxidase [*Arabidopsis thaliana*] (NP_172267) | -1.263772 | 0.416454 |
|  | Aa548 | peroxidase 52 [*Arabidopsis thaliana*] (NP_196153) | -2.091811 | 0.234586 |
|  | Aa550 | *Artemisia annua* putative calmodulin mRNA, complete cds (EF549582) | -0.793750 | 0.576843 |
|  | Aa556 | vacuolar-processing enzyme alpha-isozyme [*Arabidopsis thaliana*] (NP_180165) | -2.223755 | 0.214083 |
|  | Aa557 | *Artemisia annua* amorpha-4,11-diene monooxygenase (cyp71av1) mRNA, complete cds (DQ315671) | -1.320268 | 0.400460 |
|  | Aa561 | *Artemisia annua* P450 monooxygenase (CYP71) mRNA, complete cds (DQ667171) | -2.119147 | 0.230183 |
|  | Aa566 | ketol-acid reductoisomerase [*Arabidopsis thaliana*] (BAD94384) | -0.811984 | 0.569598 |
|  | Aa574 | chalcone isomerase [*Arabidopsis thaliana*] (AAA32766) | -1.631408 | 0.322773 |
|  | Aa576 | cinnamate-4-hydroxylase [*Arabidopsis thaliana*] (CAP08828) | -1.141436 | 0.453308 |
|  | Aa578 | Flavonoid 3'-monooxygenase [*Arabidopsis thaliana*] (NP_196416) | -1.444084 | 0.367525 |
|  | Aa580 | cytochrome P450, family 72, subfamily A, polypeptide 7 [*Arabidopsis thaliana*] (NP_188079) | -2.446256 | 0.183486 |
|  | Aa582 | cytochrome P450 81F1 [*Arabidopsis thaliana*] (AAL69519) | -3.762036 | 0.073708 |
|  | Aa583 | cytochrome like protein [*Arabidopsis thaliana*] (CAB16768) | -1.284720 | 0.410451 |
|  | Aa584 | cytochrome P450, family 81, subfamily D, polypeptide 2 [*Arabidopsis thaliana*] (NP_195452) | -2.920529 | 0.132079 |
|  | Aa586 | cytochrome P450 [*Arabidopsis thaliana*] (BAD93937) | -0.637402 | 0.642870 |
|  | Aa589 | cytochrome P450, family 82, subfamily C, polypeptide 2 [*Arabidopsis thaliana*] (NP_194925) | -2.586760 | 0.166459 |
|  | Aa591 | cytochrome P450, family 82, subfamily C, polypeptide 3 [*Arabidopsis thaliana*] (NP_194923) | -0.907154 | 0.533236 |
|  | Aa592 | cytochrome P450, family 82, subfamily G, polypeptide 1 [*Arabidopsis thaliana*] (NP_189154) | -4.440151 | 0.046066 |
|  | Aa593 | No significant similarity | -0.712852 | 0.610113 |
|  | Aa627 | uncharacterized protein [*Arabidopsis thaliana*] (NP_001154473) | -2.758683 | 0.147759 |
|  | Aa633 | cytochrome c oxidase subunit 3 [*Arabidopsis thaliana*] (NP_178782) | -0.963245 | 0.512902 |
|  | Aa634 | ATPase subunit 6 [*Arabidopsis thaliana*] (P92547) | -2.337004 | 0.197921 |
|  | Aa635 | ethylene-responsive transcription factor 9 [*Arabidopsis thaliana*] (NP_199234) | -1.199513 | 0.435422 |
|  | Aa638 | RAB geranylgeranyl transferase alpha subunit 1 [*Arabidopsis thaliana*] (NP_194180) | -0.603717 | 0.658056 |
|  | Aa649 | protein TIFY 10A [*Arabidopsis thaliana*] (NP_973862) | -0.900223 | 0.535804 |
|  | Aa653 | cytochrome c oxidase subunit 1 [*Arabidopsis thaliana*] (NP_085587) | -1.424429 | 0.372567 |
|  | Aa658 | No significant similarity | -1.815483 | 0.284109 |
|  | Aa669 | chlorophyll b synthase [*Arabidopsis thaliana*] (BAA82484) | -0.625898 | 0.648017 |
|  | Aa674 | ubiquitin family protein [*Arabidopsis thaliana*] (NP_568868) | -0.599798 | 0.659847 |
|  | Aa677 | Unknown protein [*Arabidopsis thaliana*] (AAL32564) | -0.936743 | 0.522411 |
|  | Aa678 | F-box protein 7 [*Arabidopsis thaliana*] (NP_564150) | -0.822846 | 0.565326 |
|  | Aa680 | nonspecific lipid-transfer protein precursor-like protein [*Arabidopsis thaliana*] (AAM66088) | -0.817017 | 0.567614 |
|  | Aa689 | *Artemisia annua* 3-hydroxy-3-methylglutaryl-coenzyme A reductase 2 (AAA68965.1) | -0.999420 | 0.500201 |
|  | Aa691 | retroelement pol polyprotein-like [*Arabidopsis thaliana*] (BAB10790) | -1.009578 | 0.496692 |
|  | Aa694 | mannose-binding lectin-like protein [*Arabidopsis thaliana*] (NP_849691) | -1.962402 | 0.256601 |
|  | Aa700 | Cox19-like CHCH family protein [*Arabidopsis thaliana*] (NP_196519) | -0.715507 | 0.608991 |
|  | Aa701 | VQ motif-containing protein [*Arabidopsis thaliana*] (NP_001117300) | -0.986404 | 0.504734 |
|  | Aa702 | AAA ATPase containing von Willebrand factor type A domain-containing protein [*Arabidopsis thaliana*] (NP_176883) | -1.620919 | 0.325128 |
|  | Aa703 | cytochrome c oxidase subunit 3 [*Arabidopsis thaliana*] (P92514) | -0.631476 | 0.645516 |
|  | Aa709 | phospholipid hydroperoxide glutathione peroxidase-like protein [*Arabidopsis thaliana*] (BAA24226) | -1.471459 | 0.360617 |
|  | Aa711 | S-adenosylmethionine synthetase [*Arabidopsis thaliana*] (AAA32868) | -2.396271 | 0.189955 |
|  | Aa714 | inorganic carbon transport protein-related protein [*Arabidopsis thaliana*] (NP_177233) | -1.022893 | 0.492129 |
|  | Aa717 | N-glyceraldehyde-2-phosphotransferase-like [*Arabidopsis thaliana*] (BAA97552) | -1.169467 | 0.444586 |
|  | Aa731 | L-ascorbate oxidase [*Arabidopsis thaliana*] (NP_001154729) | -4.713993 | 0.038102 |
|  | Aa732 | ATP sulfurylase like protein [*Arabidopsis thaliana*] (BAD95100) | -1.616018 | 0.326235 |
|  | Aa733 | mraW methylase family protein [*Arabidopsis thaliana*] (NP_196652) | -0.676481 | 0.625689 |
|  | Aa741 | hypothetical protein [*Arabidopsis thaliana*] (BAD93976) | -2.299193 | 0.203177 |
|  | Aa742 | putative serine/threonine kinase [*Arabidopsis thaliana*] (BAC43390) | -1.658330 | 0.316806 |
|  | Aa745 | Exosome complex component RRP4 [*Arabidopsis thaliana*] (NP_171835) | -2.140791 | 0.226755 |
|  | Aa747 | S-adenosyl-L-methionine-dependent methyltransferase-like protein [*Arabidopsis thaliana*] (NP_176478) | -1.808345 | 0.285518 |
|  | Aa755 | NmrA-like negative transcriptional regulator family protein [*Arabidopsis thaliana*] (NP_195634) | -2.839433 | 0.139716 |
|  | Aa756 | Isoflavone reductase-P3 [*Arabidopsis thaliana*] (NP_565107) | -0.870179 | 0.547079 |
|  | Aa757 | NmrA-like negative transcriptional regulator family protein [*Arabidopsis thaliana*] (NP_195634) | -1.728272 | 0.301813 |

Criteria for the comparative analysis adopted here was that genes with log fold change <= -0.5849 (FC <= 0.66) were declared as down-regulated in seedling, whereby mature plant leaf sample was taken as the control.
