# Supplementary material for: Differentially Expressed Genes during Contrasting Growth Stages of Artemisia annua for Artemisinin Content
Source: PLoS One. 2013 Apr 3;8(4):e60375. doi: 10.1371/journal.pone.0060375 (PMC3616052; doi:10.1371/journal.pone.0060375)
Supplement: Table S5 — Genes found to be up-regulated in seedling as compared to mature plant leaf in A. annua . (DOC) [file pone.0060375.s007.doc]

**Table S5:** Genes found to be up-regulated in seedling as compared to mature plant leaf in *Artemisia annua*.

| **S. No.** | **Gene / Probe ID** | **Gene / Probe Homology-based Annotation / Description** | **Log Fold Change** | **Fold Change** |
| --- | --- | --- | --- | --- |
|  | Aa6 | ubiquinol--cytochrome-c reductase-like protein [*Arabidopsis thaliana*] (AAM62600) | 0.786647 | 1.725061 |
|  | Aa19 | cytochrome P450, family 51 (sterol 14-demethylase) [*Arabidopsis thaliana*] (NP_172633) | 0.968547 | 1.956868 |
|  | Aa28 | cytochrom P450-like protein [*Arabidopsis thaliana*] (AAM66094) | 2.027971 | 4.078310 |
|  | Aa31 | cytochrome P450, family 77, subfamily B, polypeptide 1 [*Arabidopsis thaliana*] (NP_172626) | 1.060499 | 2.085653 |
|  | Aa36 | cytochrome P450 homolog [*Arabidopsis thaliana*] (CAB45979) | 0.836455 | 1.785657 |
|  | Aa37 | cytochrome P450, family 72, subfamily A, polypeptide 8 [*Arabidopsis thaliana*] (NP_188080) | 1.193271 | 2.286707 |
|  | Aa58 | Cytochrome b-c1 complex, subunit 8 protein [*Arabidopsis thaliana*] (NP_196156) | 0.675756 | 1.597433 |
|  | Aa67 | putative cytochrome P450 [*Arabidopsis thaliana*] (NP_001117558) | 1.102678 | 2.147530 |
|  | Aa71 | cytochrome P450, family 706, subfamily A, polypeptide 3 [*Arabidopsis thaliana*] (NP_199275) | 0.628730 | 1.546203 |
|  | Aa74 | cytochrome like protein [*Arabidopsis thaliana*] (CAB16768) | 0.844030 | 1.795058 |
|  | Aa79 | cytochrome P450, family 86, subfamily A, polypeptide 4 [*Arabidopsis thaliana*] (NP_171666) | 0.857529 | 1.811932 |
|  | Aa84 | cytochrome P450, family 81, subfamily D, polypeptide 8 [*Arabidopsis thaliana*] (NP_195453) | 2.064044 | 4.181568 |
|  | Aa94 | nuclear transcription factor Y subunit C-1 [*Arabidopsis thaliana*] (NP_190428) | 1.270854 | 2.413043 |
|  | Aa106 | tubby-like F-box protein 1 [*Arabidopsis thaliana*] (NP_177816) | 0.584985 | 1.500023 |
|  | Aa112 | E2F transcription factor 3 [*Arabidopsis thaliana*] (NP_565831) | 0.741585 | 1.672011 |
|  | Aa114 | tubby-like F-box protein 10 [*Arabidopsis thaliana*] (NP_173899) | 0.838664 | 1.788393 |
|  | Aa127 | AP2/ERF and B3 domain-containing transcription factor RAV1 [*Arabidopsis thaliana]* (NP_172784) | 1.283591 | 2.434441 |
|  | Aa130 | plastid transcriptionally active 14 protein [*Arabidopsis thaliana*] (NP_193746) | 0.865992 | 1.822592 |
|  | Aa133 | protein plastid transcriptionally active 16 [*Arabidopsis thaliana*] (NP_566886) | 0.804669 | 1.746745 |
|  | Aa140 | protein brassinazole-resistant 2 [*Arabidopsis thaliana*] (NP_973863) | 0.694180 | 1.617964 |
|  | Aa150 | RGA1 protein [*Arabidopsis thaliana*] (CAA72177) | 1.179778 | 2.265420 |
|  | Aa158 | indoleacetic acid (IAA)-inducible gene (IAA7) [*Arabidopsis thaliana*] (AAM65301) | 1.595321 | 3.021618 |
|  | Aa177 | transcription factor MYC2 [*Arabidopsis thaliana*] (NP_174541) | 0.645030 | 1.563771 |
|  | Aa185 | transcription factor AS1 [*Arabidopsis thaliana*] (NP_181299) | 1.214433 | 2.320496 |
|  | Aa195 | scarecrow-like 5 [*Arabidopsis thaliana*] (AAD24405) | 0.819845 | 1.765217 |
|  | Aa209 | zinc finger protein STZ/ZAT10 [*Arabidopsis thaliana*] (NP_174094) | 0.671444 | 1.592667 |
|  | Aa216 | ethylene-responsive transcription factor ERF025 [*Arabidopsis thaliana*] (NP_200015) | 1.396486 | 2.632596 |
|  | Aa284 | Preprotein translocase subunit secY [*Arabidopsis thaliana*] (NP_179461) | 0.629758 | 1.547305 |
|  | Aa286 | inner membrane protein ALBINO3 [*Arabidopsis thaliana*] (NP_001118405) | 0.665693 | 1.586330 |
|  | Aa291 | Nucleotide-sugar transporter family protein [*Arabidopsis thaliana*] (NP_192719) | 0.788168 | 1.726880 |
|  | Aa296 | auxin efflux carrier component 3 [*Arabidopsis thaliana*] (NP_177250) | 0.965973 | 1.953381 |
|  | Aa307 | ribonucleotide reductase small subunit [*Arabidopsis thaliana*] (Q6Y657) | 0.620296 | 1.537190 |
|  | Aa308 | *Artemisia annua* 3-hydroxy-3-methylglutaryl coenzyme A reductase (AHM4) mRNA, (U14625) | 0.788549 | 1.727336 |
|  | Aa321 | putative protein [*Arabidopsis thaliana*] (CAA19721) | 0.765039 | 1.699416 |
|  | Aa322 | enoyl-ACP reductase [*Arabidopsis thaliana*] (CAA74175) | 1.145828 | 2.212731 |
|  | Aa327 | short chain alcohol dehydrogenase-like [*Arabidopsis thaliana*] (AAM61117) | 0.617847 | 1.534583 |
|  | Aa330 | Rossmann-fold NAD(P)-binding domain-containing protein [*Arabidopsis thaliana]* (NP_849428) | 0.759078 | 1.692408 |
|  | Aa332 | uncharacterized protein [*Arabidopsis thaliana*] (NP_182212) | 0.729532 | 1.658102 |
|  | Aa346 | Rossmann-fold NAD(P)-binding domain-containing protein [*Arabidopsis thaliana*] (NP_849428) | 1.087424 | 2.124942 |
|  | Aa360 | Oxidoreductase family protein [*Arabidopsis thaliana*] (NP_193468) | 0.611671 | 1.528028 |
|  | Aa361 | NAD(P)-linked oxidoreductase-like protein [*Arabidopsis thaliana*] (NP_171937) | 0.628883 | 1.546367 |
|  | Aa366 | putative NADH dehydrogenase [ubiquinone] [*Arabidopsis thaliana*] (NP_566192) | 0.713490 | 1.639766 |
|  | Aa368 | Rossmann-fold NAD(P)-binding domain-containing protein [*Arabidopsis thaliana*] (NP_849428) | 0.716330 | 1.642997 |
|  | Aa376 | phosphoadenosine phosphosulfate (PAPS) reductase family protein [*Arabidopsis thaliana*] (BAF00583) | 0.722509 | 1.650049 |
|  | Aa378 | putative dehydrogenase [*Arabidopsis thaliana*] (AAM65725) | 0.715935 | 1.642547 |
|  | Aa383 | NADH dehydrogenase (ubiquinone) 1 alpha subcomplex 8 [*Arabidopsis thaliana]* (NP_566280) | 0.736315 | 1.665915 |
|  | Aa384 | beta-oxoacyl-(acyl carrier protein) reductase [*Arabidopsis thaliana*] (AAC00590) | 0.746868 | 1.678146 |
|  | Aa402 | UDP-XYL synthase 5 [*Arabidopsis thaliana*] (NP_190228) | 0.749213 | 1.680876 |
|  | Aa421 | naphthoate synthase [*Arabidopsis thaliana*] (NP_176255) | 0.783317 | 1.721084 |
|  | Aa436 | tryptophan synthase alpha chain [*Arabidopsis thaliana*] (NP_192170) | 1.074609 | 2.106151 |
|  | Aa445 | 1-aminocyclopropane-1-carboxylate synthase 6 [*Arabidopsis thaliana*] (NP_192867) | 1.114773 | 2.165609 |
|  | Aa469 | dihydrolipoyl dehydrogenase 1 [*Arabidopsis thaliana*] (NP_175237) | 0.768385 | 1.703362 |
|  | Aa476 | dihydrolipoyllysine-residue succinyltransferase component of 2-oxoglutarate dehydrogenase complex 1 [*Arabidopsis thaliana*] (NP_200318) | 1.087914 | 2.125665 |
|  | Aa479 | UDP-glucose dehydrogenase, putative [*Arabidopsis thaliana*] (AAM67208) | 0.759153 | 1.692497 |
|  | Aa482 | NADH dehydrogenase subunit K [*Arabidopsis thaliana*] (NP_051063) | 0.669207 | 1.590198 |
|  | Aa486 | 2-oxoglutarate dehydrogenase, E1 subunit - like protein [*Arabidopsis thaliana*] (BAF01713) | 0.856046 | 1.810070 |
|  | Aa488 | UDP-glucose dehydrogenase, putative [*Arabidopsis thaliana*] (AAM67208) | 0.959374 | 1.944466 |
|  | Aa491 | 2-oxoisovalerate dehydrogenase E1 component, beta subunit [*Arabidopsis thaliana*] (NP_187954) | 0.725207 | 1.653138 |
|  | Aa499 | aldehyde dehydrogenase 3F1 [*Arabidopsis thaliana*] (NP_195348) | 0.608138 | 1.524291 |
|  | Aa503 | semialdehyde dehydrogenase-like protein [*Arabidopsis thaliana*] (NP_172934) | 0.610082 | 1.526346 |
|  | Aa508 | pyruvate dehydrogenase E1 component subunit alpha [*Arabidopsis thaliana*] (NP_171617) | 1.412230 | 2.661482 |
|  | Aa541 | peroxidase 52 [*Arabidopsis thaliana*] (NP_196153) | 0.691506 | 1.614969 |
|  | Aa545 | peroxidase 52 [*Arabidopsis thaliana*] (NP_196153) | 0.593086 | 1.508470 |
|  | Aa547 | peroxidase ATP24a [*Arabidopsis thaliana*] (CAA72484) | 1.827388 | 3.548940 |
|  | Aa549 | actin 7 [*Arabidopsis thaliana*] (NP_196543) | 0.746753 | 1.678012 |
|  | Aa554 | *Artemisia annua* 1-deoxy-D-xylulose-5-phosphate reductoisomerase (DXR1) mRNA, complete cds (AF182287) | 1.349311 | 2.547904 |
|  | Aa555 | *Artemisia annua* mRNA for putative sesquiterpene cyclase (cASC125 gene) (AJ271792) | 1.543833 | 2.915681 |
|  | Aa569 | putative DNA gyrase subunit B [*Arabidopsis thaliana*] (AAF02815) | 0.785690 | 1.723917 |
|  | Aa590 | cytochrome P450, family 77, subfamily B, polypeptide 1 [*Arabidopsis thaliana*] (NP_172626) | 1.200530 | 2.298241 |
|  | Aa600 | maturase K [*Arabidopsis thaliana*] (AAG43340) | 1.900507 | 3.733445 |
|  | Aa718 | phosphoenolpyruvate carboxykinase [ATP] [*Arabidopsis thaliana*] (NP_195500) | 0.779621 | 1.716679 |
|  | Aa738 | catalase 2 [*Arabidopsis thaliana*] (NP_001031791) | 1.093858 | 2.134441 |
|  | Aa752 | inorganic phosphate transporter 2-1 [*Arabidopsis thaliana*] (NP_850633) | 0.776489 | 1.712958 |

Criteria for the comparative analysis adopted here was that genes with log fold change value >= 0.5849 (FC >= 1.5) were declared as up-regulated in seedling, whereby mature plant leaf sample was taken as the control.
